# Spatial Control of 2D Nanomaterial Electronic Properties Using Chiral Light Beams

**DOI:** 10.1021/acsnano.4c04506

**Published:** 2024-07-29

**Authors:** Paula L. Lalaguna, Paul Souchu, Neel Mackinnon, Frances Crimin, Rahul Kumar, Shailendra Kumar Chaubey, Asma Sarguroh, Amy McWilliam, Alexey Y. Ganin, Donald A. MacLaren, Sonja Franke-Arnold, Jörg B. Götte, Stephen M. Barnett, Nikolaj Gadegaard, Malcolm Kadodwala

**Affiliations:** † School of Chemistry, University of Glasgow, Glasgow G12 8QQ, U.K.; ‡ Faculté des sciences et ingénierie, Université de Toulouse UPS, Toulouse 31400, France; § SUPA, School of Physics and Astronomy, University of Glasgow, Glasgow G12 8QQ, U.K.; ∥ James Watt School of Engineering, University of Glasgow, Glasgow G12 8QQ, U.K.

**Keywords:** chirality, orbital angular momentum, strain, two-dimensional materials, graphene, TMDCs

## Abstract

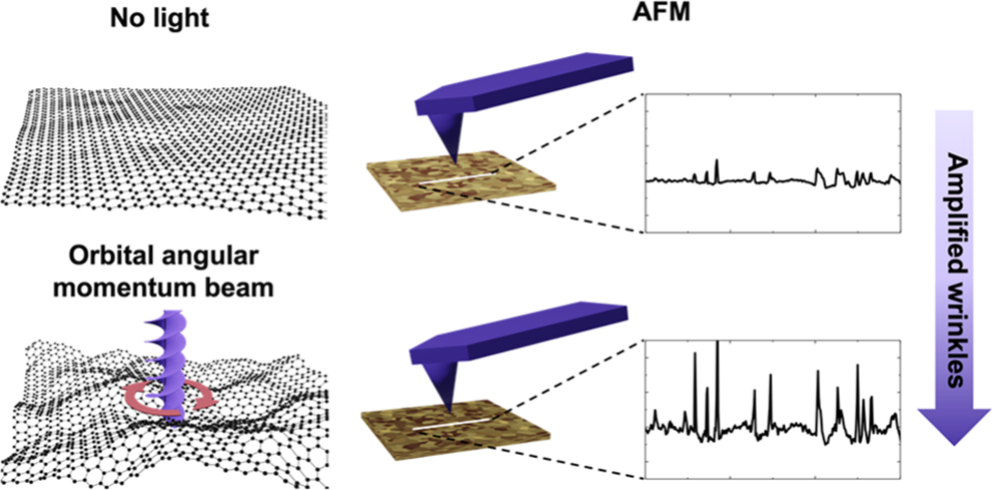

Single-layer two-dimensional (2D) nanomaterials exhibit physical and chemical properties which can be dynamically modulated through out-of-plane deformations. Existing methods rely on intricate micromechanical manipulations (*e.g*., poking, bending, rumpling), hindering their widespread technological implementation. We address this challenge by proposing an all-optical approach that decouples strain engineering from micromechanical complexities. This method leverages the forces generated by chiral light beams carrying orbital angular momentum (OAM). The inherent sense of twist of these beams enables the exertion of controlled torques on 2D monolayer materials, inducing tailored strain. This approach offers a contactless and dynamically tunable alternative to existing methods. As a proof-of-concept, we demonstrate control over the conductivity of graphene transistors using chiral light beams, showcasing the potential of this approach for manipulating properties in future electronic devices. This optical control mechanism holds promise in enabling the reconfiguration of devices through optically patterned strain. It also allows broader utilization of strain engineering in 2D nanomaterials for advanced functionalities in next-generation optoelectronic devices and sensors.

## Introduction

Two-dimensional (2D) materials, such as graphene and transition metal dichalcogenides (TMDCs), exhibit extraordinary properties with applications spanning from advanced electronics to energy storage.^1−3^ Despite their high tensile strengths and Young’s moduli,^4^ 2D materials can undergo out-of-plane deformation with minimal strain (<10^–12^ N),^5,6^ which has been shown to influence key properties such as electron mobility, band gaps, chemical reactivity and thermal conductivity.^7−10^ In principle, modulating out-of-plane deformations could therefore provide a tunable, reconfigurable control of electronic properties. However, such dynamic manipulation has previously required nano/micromechanical actuators that increase device complexity and limit the prospect for reconfigurability. Here, we demonstrate the concept of optical strain engineering of 2D materials using chiral light beams that possess orbital angular momentum (OAM). Illumination with OAM is shown to induce shear strains in two prototypical 2D materials, graphene and monolayer WS_2_, producing measurable changes in their electronic characteristics. Critically, this control is both dynamic and reversible.

**Figure 1:**
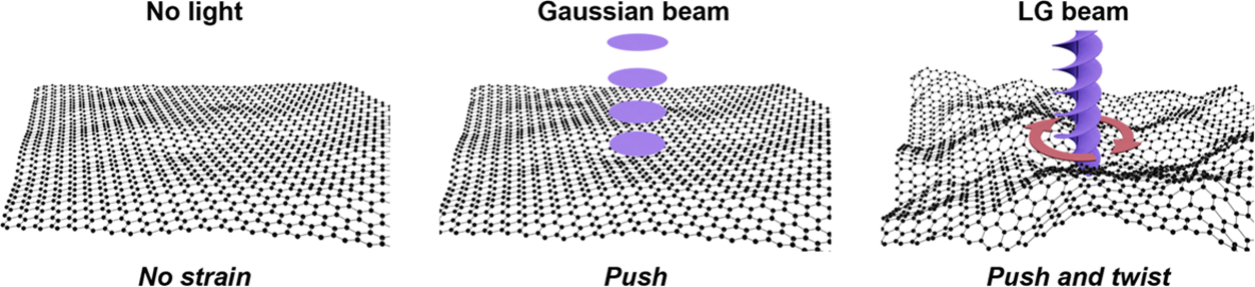
Concept of strain engineering of 2D materials using light beams. In the absence of a light beam, the material remains unperturbed. A slight increase in roughness is caused by the linear momentum carried by a conventional Gaussian beam (push). LG beams carrying OAM also cause shear strain (push and twist), leading to substantial out-of-plane deformations.

Our work exploits the orbital angular momentum (OAM) carried by Laguerre-Gaussian (LG) beams. These beams possess helical phase fronts, which endow them with the property of chirality, and carry OAM that is quantized to ± *l*ℏ per photon, where the quantum number *l* is referred to as the topological charge.^11^ The application of angular momentum from the LG beam to a monolayer produces shear strains that induce morphological changes, as shown schematically in Figure 1. LG beams are already commonly used for the optomechanical manipulation of matter at the mesoscale, most famously for optical tweezing, where transfer of orbital angular momentum from light to an object induces rotation about the beam axis.^12−17^ In other experiments, mesoscopic helical polymers and metal nanoneedles have been sculpted by mass transport of molten material during illumination with an LG beam.^18−20^ In this work we demonstrate a fundamentally different application of OAM: the reversible induction of shear strain in 2D materials, offering a dynamic control of the electrical and optical properties. This proof-of-principle demonstration has wide-ranging implications for optomechanical manipulation of the electronic functionality of 2D materials.

## Results and Discussion

### Monolayer Graphene

**Figure 2:**
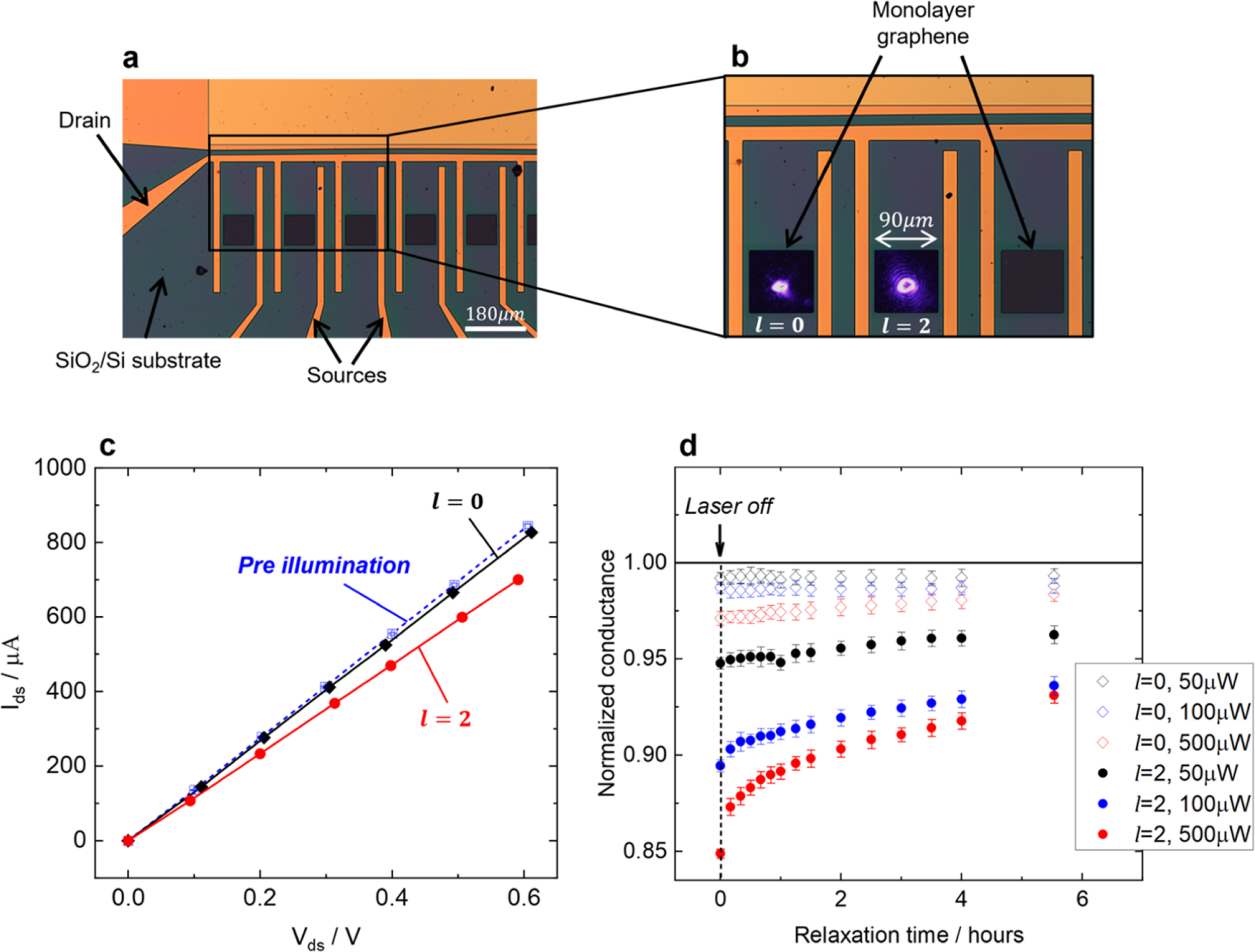
Effects of OAM on the conductance of CVD monolayer graphene. (a) Optical microscopy image of the GFET used for the experiments. The GFET includes several monolayer graphene channels connected to a common drain and individual sources, where each graphene channel can be selected with external individual source switches. (b) Inset of (a) with an *l* = 0 beam on a monolayer graphene channel and *l* = 2 beam on a different channel. (c) *I*–*V* characteristics after a pristine monolayer graphene is illuminated with *l* = 0 (black) and *l* = 2 (red) compared to prior to illumination (blue). Excitation power: 500 μW. (d) Relaxation of the conductance as a function of time once the laser is switched off after illuminating monolayer graphene with *l* = 0 (unfilled diamonds) and *l* = 2 (filled circles) at 50 μW (black), 100 μW (blue) and 500 μW(red). The conductance has been normalized with that prior to laser illumination (black horizontal line).

A commercially available graphene-FET (GFET) was used as an exemplar system to illustrate how shear strain generated by LG beams can be used to reconfigure the response of 2D material-based electronic devices. The electrical properties of graphene are known to be highly dependent on strain induced by out-of-plane deformations^7,21^ and conductivity measurements provide a direct measurement of the influence of strain, with wrinkles and rumples reducing the conductance.^7,21^ The active component of the GFET is a 90 μm × 90 μm CVD (chemical vapor deposition)-grown monolayer graphene (Methods), illustrated in Figure 2a,b. The graphene sheet was illuminated with a ∼30 μm diameter spot (Figure S3) of either a Gaussian (*l* = 0) or LG (*l* = 2) beam (λ_ex_ = 405 nm). In both cases, the experiment was performed with linearly polarized light under ambient conditions and the conductance was monitored through the collection of *I*–*V* curves (see Methods). Illumination with both Gaussian and LG beams caused a reduction in the conductance; however, the LG beams induced a significantly larger reduction (Figure 2c,d). The level of conductance reduction also correlates with the power of the beam (Figure 2d), which can be explained as the magnitude of the optical forces and torques is directly proportional to laser power. The reduction in conductance is consistent with the observations of a previous study where conductance as a function of out-of-plane (bending) strain was monitored.^21^

Once the laser illumination was switched off, relaxation of the conductance back to its initial value occurred over several hours (Figure 2d). The relaxation curve could not be fitted to a single exponential decay function, indicating a multistep relaxation process. For both Gaussian and LG illumination and for all laser powers, the conductance returned to >90% of its preillumination value within 6 hours (Figure 2d). Complete relaxation to the preilluminated conductance took up to ∼48 hours. We propose an intuitive model to explain the apparent bimodal relaxation process of graphene, characterized by both rapid and slower phases. We suggest that the slower relaxation process is associated with wrinkles “pinned” to defects in the underlying substrate. The activation barrier for the relaxation of such pinned wrinkles is higher compared to those formed in defect-free regions of the substrate.^22^ Consequently, pinned wrinkles relax more slowly than those on defect-free regions.

**Figure 3:**
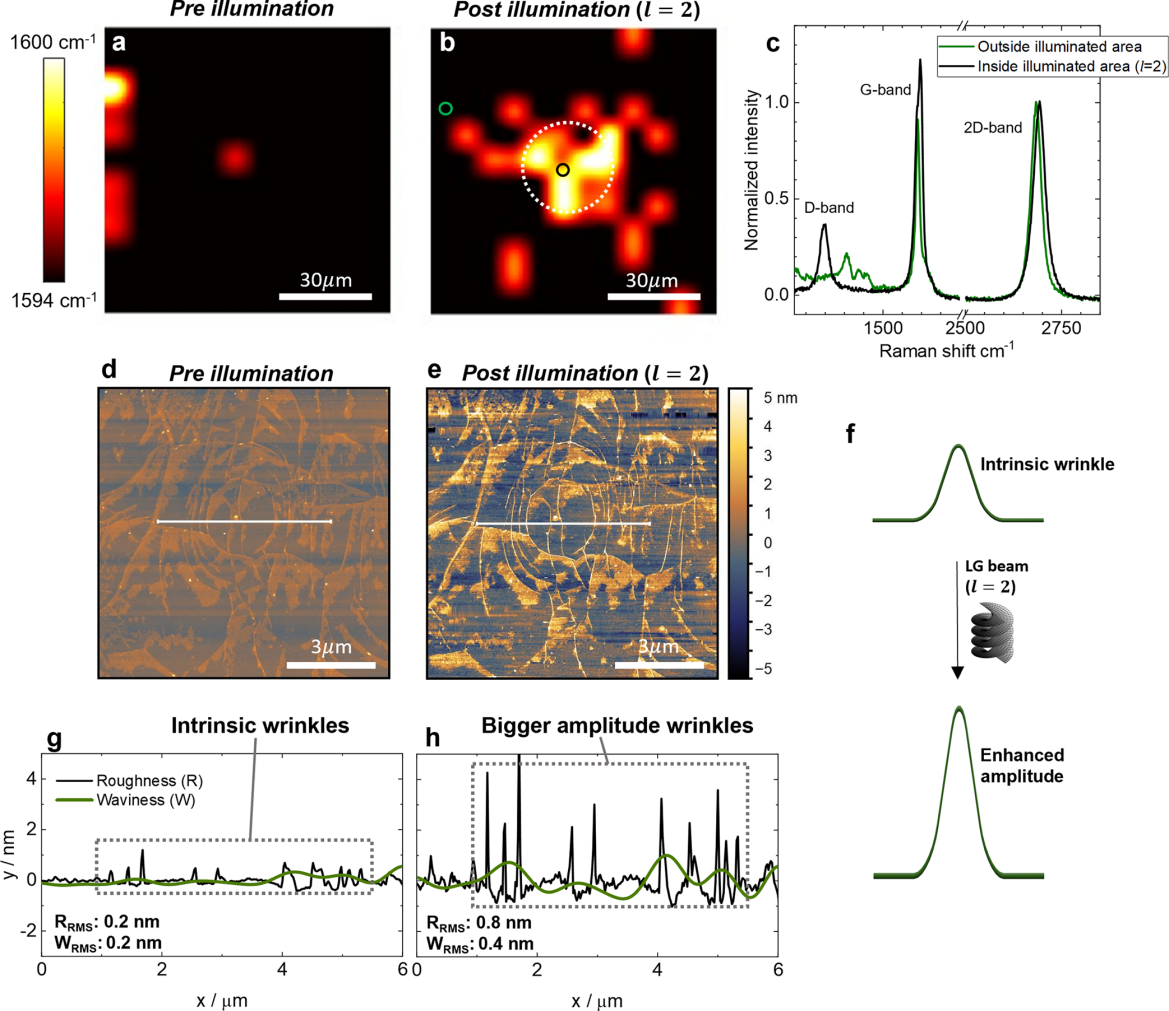
OAM-induced wrinkles in CVD monolayer graphene. (a, b) Raman mapping of the G-band before (a) and after LG beam (*l* = 2) illumination (b), showing a blue shift at the illuminated central area (scale bar: 30 μm). The approximate position of the illuminated area in panel (b) is indicated with a white dotted circle. (c) Raman spectra for illuminated (black) and unilluminated (green) areas collected at the positions depicted by the black and green circles, respectively, in panel (b). The spectra have been normalized to the maximum intensity of the 2D-band. (d, e) AFM characterization of monolayer graphene before illumination (d) and after illumination (e) with an LG (*l* = 2) beam (scale bar: 3 μm). The root-mean-square roughness (*R*_RMS_) before illumination in (d) is 0.4 nm, and after LG beam illumination in (e) is 1.5 nm. (f) Schematic of an intrinsic wrinkle whose amplitude increases after illumination with an LG beam. (g, h) Variation in roughness (black) and waviness (green) across the white cut lines in (d) and (e), respectively. The root-mean-square roughness (*R*_RMS_) and waviness (*W*_RMS_) have been added at the bottom left corner (see Methods for details).

We used Raman mapping to verify that the changes in the electrical response of the GFET are the result of local physical modifications of the structure of graphene. The Raman spectra of monolayer graphene are dominated by two peaks, commonly referred to as G- (1590 cm^–1^) and 2D- (2685 cm^–1^) bands.^23^ For well-ordered, flat monolayer graphene the G-band is less intense than the 2D-band.^24,25^ Disorder in graphene can be defined as a physical modification that effectively reduces translation symmetry, including the formation of wrinkles.^25^ The presence of disorder is signaled by a definitive spectral fingerprint, with the appearance of a new peak referred to as the D-band (1350 cm^–1^).^23^ The Raman map was collected using the G-band wavelength for the 90 μm × 90 μm monolayer graphene of the GFET and shows that before illumination there is some spatial heterogeneity (Figure 3a). Raman maps were collected after LG beam illumination and a blue shift is observed at the illuminated area (lying in the center of Figure 3b), which is attributed to the development of strain in the sample.^26^ In addition, the illuminated area shows the appearance of the D-band at 1350 cm^–1^ and an increase of the G/2D intensity ratio (Figure 3c), which has previously been attributed to the formation of wrinkles.^24,25,27^ These observations are consistent with our conductance measurements, as wrinkles are also known to decrease the electrical conductivity of graphene.^7,28^ After a relaxation period >48 hours, the monolayer graphene relaxed back to a more ordered (*i*.*e*. less wrinkly) state. Specifically, the D-band is almost indistinguishable and the G/2D ratio is less than 1 (see Figure S4). Equivalent Raman maps collected with a Gaussian beam show no indication of an increase in the G/2D ratio or a prominent appearance of the D-band (Figure S5).

We used atomic force microscopy (AFM) to measure morphological changes of graphene after illumination with LG beams. The AFM images were taken from an equivalent monolayer graphene rather than from the GFET due to experimental constraints (Methods). The AFM image collected prior to illumination is consistent with those observed previously from CVD-grown graphene.^7,29,30^ It shows intrinsic wrinkles which manifest as a distinct pattern of bright lines in the AFM image, highlighted in Figure 3d, and appear to be <2 nm high (Figure 3g). Illumination with the LG beam does not significantly change the pattern of the wrinkling (*i*.*e*., the number of wrinkles does not alter dramatically). However, there is a notable increase in the height of the pre-existing wrinkles (Figure 3e,h). The increase in the height of wrinkles induced by LG illumination can be parameterized by the root-mean-square roughness (*R*_RMS_) of the imaged area. Illumination of the area with an LG beam results in an increase in *R*_RMS_ of 1.1 nm. Illumination with a conventional Gaussian beam has significantly smaller effects on the morphology of the graphene, with no significant increase in the amplitude of the wrinkles (Figure S6). Thus, the AFM images show the greater effectiveness of LG beam to induce larger wrinkles, and hence rationalizes the electrical responses of the GFET.

A thermal origin for the reduction in conductance can be unambiguously ruled out. The relaxation time is too long for a thermal effect, but more importantly, the conductance of graphene is known to increase with temperature.^31^ Furthermore, heating CVD-grown graphene on SiO_2_/Si has previously been shown to lead to unwrinkling of graphene^29^ and red shift of the G-band,^32^ contrary to our observations. For completeness, numerical simulations (Methods) were used to quantify temperature rises induced by Gaussian and LG beams. The modeling (Figure S9) shows that Gaussian beams induced the largest temperature rises (Δ*T* = 0.77 K) in the monolayer graphene compared to LG beams (Δ*T* = 0.67 K). Consequently, we conclude that the decrease in conductance and increased roughness are associated with the strain induced in graphene by optical forces produced by both Gaussian and LG beams. Furthermore, the torque imparted by LG beams significantly enhances the effect.

### Monolayer WS_2_

To further illustrate how 2D materials can be manipulated using optical strain engineering, and the influence of the nature of the monolayer-substrate interface, the effects of LG beams on exfoliated monolayer WS_2_ flakes and full-coverage CVD-grown monolayer WS_2_ have been studied. The exfoliated monolayer flakes had a range of lateral dimensions approximately spanning 5–40 μm and were deposited on a SiO_2_/Si substrate. The CVD-grown monolayer WS_2_ (1 cm × 1 cm) was grown on a sapphire substrate (Methods). Monolayer WS_2_ is a direct-band gap semiconductor and it exhibits strong photoluminescence (PL).^33^ PL spectroscopy is routinely used to characterize the properties of monolayer WS_2_^34,35^ and out-of-plane deformations, such as wrinkling, lead to a red shift in the PL spectra.^6,8,9,36−39^ PL spectroscopy was therefore used to monitor the level of strain induced in monolayer WS_2_. In general, PL spectra collected from WS_2_ monolayers have components associated with neutral (X^0^) and trionic (T) excitonic states. Trions are formed when an electron or a hole bind to a neutral exciton.^40^

**Figure 4:**
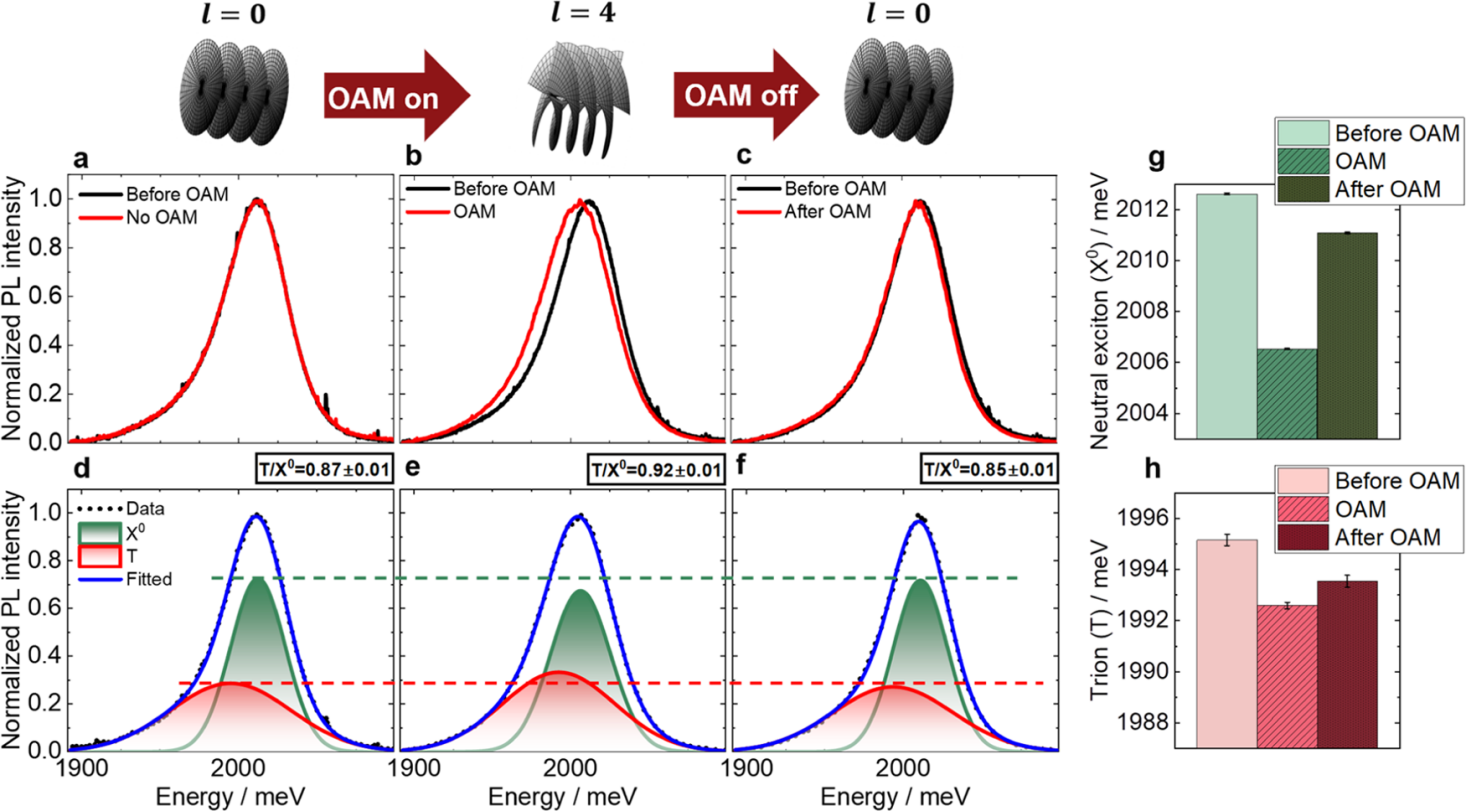
OAM-induced red shift in the PL spectrum of CVD monolayer WS_2_. (a) Reference spectra with no OAM (red) and right before OAM is introduced (black), showing no change between the two consecutive spectra. (b) Spectrum collected with OAM *l* = 4 (red) compared to the spectrum before OAM (black). (c) Spectrum collected with a Gaussian beam after OAM (red), compared to the spectrum before OAM (black). (d–f) Gaussian fits for the red spectra shown in (a–c), respectively. The green and red horizontal lines indicate changes in neutral exciton and trion emissions, respectively, and the T/X^0^ intensity ratio is indicated at the top right corner. (g, h) Changes in the energies of the neutral exciton X^0^ (g) and trion T (h) obtained from the fittings shown in panels (d–f).

The induction of strain in WS_2_ was inferred from differences in PL spectra collected during illumination with LG and Gaussian beams. For brevity, only the data for CVD-grown monolayer are discussed in detail here, while the data from exfoliated flakes can be found in Supplementary Section 7. Illumination of the CVD-grown WS_2_ with LG beams result in both a red shift in the X^0^ component and an increase in the T/X^0^ intensity ratio, as shown in Figure 4. Crucially, however, the magnitude of these effects varies across the sample for the CVD monolayer and between flakes for the exfoliated sample. The red shift in the X^0^ feature induced by the LG beam can be correlated to the initial strain state of the monolayer. Specifically, the lowest T/X^0^ intensity ratio corresponds to the minimum initial strain, and this leads to the largest red shift with the LG beam (Figure S12). An example of unnormalized spectra before and after illumination of OAM is shown in Figure S13. Note that similar effects are observed with beams of opposite handedness (*l* = −4), as shown in Figure S14. Once the beam was switched from LG to Gaussian illumination, a residual red shift in the PL spectra remained (Figure 4). This effect is most pronounced for the exfoliated sample, with the PL spectra returning to close to its initial line shape after 2 hours (Figure S11). Previous work has demonstrated that the X^0^ component red shifts and the T/X^0^ intensity ratio increases with strain caused by out-of-plane deformations, such as wrinkles.^41^ Due to the faster relaxation times displayed by WS_2_ samples compared to graphene, wrinkling cannot be monitored directly with AFM and Raman mapping. The residual spectral changes after switching back from LG to Gaussian beams rules out the possibility of an electronic origin of the effect.^42^

A thermal origin accounting for the difference between Gaussian and LG spectra can again be ruled out. Specifically, heating the sample causes different spectral changes to those produced by an LG beam (Figures S15 and S16). Furthermore, our numerical thermal simulations (Methods) show that, under equivalent conditions, LG beams have lower power density, resulting in temperature rises ∼0.3 K smaller than a Gaussian beam (Figures S17 and S18). Additionally, PL spectra collected from CVD monolayer show no significant change over a power range going beyond that used to collect the reported data (Figure S19). Therefore, the red shift observed with the LG beam cannot be accounted for by a difference in power density between a Gaussian and LG beam.

To summarize, OAM has qualitatively similar effects on both types of WS_2_ monolayers, with PL data consistent with strain caused by the induction of wrinkles. When viewed collectively the WS_2_ and graphene data do show that the kinetics of the relaxation of the monolayers back to equilibrium states are dependent on the nature of the interfaces. This is to be expected since the frictional forces, which will govern relaxation, are strongly correlated to the physical and chemical properties of the substrate upon which the monolayers are placed.^22^ The longer relaxation time of WS_2_ on sapphire compared to silicon aligns with their typical coefficient of friction values. Silicon generally has a higher coefficient of friction (∼0.4)^43^ compared to sapphire (∼0.2).^44^

### Numerical Simulations of Optical Forces and Torques

**Figure 5:**
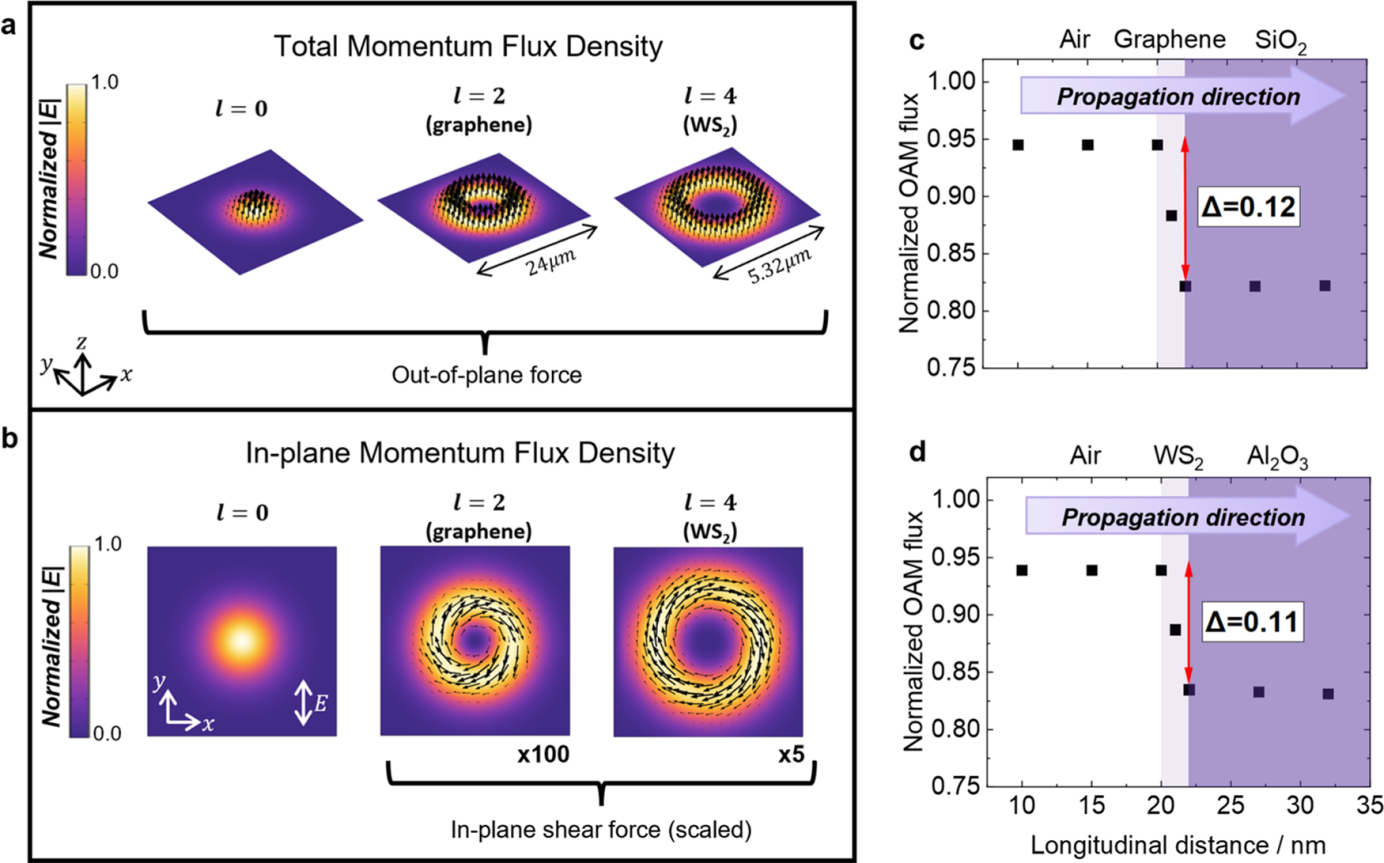
Numerical simulations of time-averaged momentum flux density and orbital angular momentum transfer to graphene and monolayer WS_2_. (a) Total momentum flux density for *l* = 0, *l* = 2 and *l* = 4 beams at the focal plane, superimposed on the normalized electric field intensity. (b) In-plane (transverse) momentum flux density for *l* = 0, *l* = 2 and *l* = 4 beams at the focal plane, superimposed on the normalized electric field intensity. The in-plane momentum flux density for graphene and WS_2_ in (b) have been scaled to match the range of the total momentum flux density in (a). (c) Simulated orbital angular momentum flux as an LG beam (*l* = 2) propagates through air, graphene and SiO_2_, normalized by the orbital angular momentum flux of the beam in air. (d) Simulated orbital angular momentum flux as an LG beam (*l* = 4) propagates through air, WS_2_ and Al_2_O_3_, normalized by the orbital angular momentum flux of the beam in air.

The wrinkling of the film is generated by a torque or lateral force arising from the action of the LG beam. We can understand the origin of the torque acting on our 2D materials simply from Newton’s second law of motion, which states that the force acting is given by the rate of change of the momentum. Equivalently, the torque arises from the rate of change of the angular momentum. Our experiment employs a continuous-wave laser and so there is no time development of the momentum density but the change in the field momentum within the material is fully determined by the momentum flux density, that is the flow of the optical momentum through the medium. As derived in Supplementary Section 11, the components of the momentum flux density through the interface are
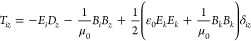
1where *i* = *x*,*y*,*z*. To map out the forces in the focal plane, the simulated time-averaged optical momentum flux density is displayed in Figure 5a,b. For the simulated linearly polarized Gaussian beam, the optical momentum flux points only out-of-plane in the direction of light propagation (Figure 5a). This qualitatively replicates the effects achieved by a physical push of monolayer WS_2_, for instance, similar to that generated by AFM tips in contact mode.^41^ For LG beams, the optical momentum flux density still has a significant out-of-plane component, but in addition there is an in-plane shear force, corresponding to the azimuthal component of the momentum flux (Figure 5b). Thus, an LG beam essentially “pushes and twists” at a monolayer, and it is this that increases the size of the wrinkles and subsequently modifies the electronic properties of the monolayers.

The forces induced by the optical momentum induce a local shear on the surface of the film and with it a torque. This torque originates from the orbital angular momentum carried by LG light beams. Thus, we can also understand the wrinkling of the film as a consequence of the flux of the angular momentum carried by the light. For LG beams propagating through WS_2_ or graphene, it is calculated (using the expression derived in Supplementary Sections 12–13) that ∼12% of the OAM flux carried by the beam is transferred to the monolayer (Figures 5c,d, and S20). In the case of graphene (405 nm, *l* = 2, 500 μW), this level of exchange of OAM flux would generate an in-plane torque of 2.1 × 10^–20^ N m,^12^ which compares to the bending rigidity of graphene of 1.3–2.6 × 10^–19^ N m, reported in previous studies.^45,46^ We believe this is why optical torques predominantly amplify pre-existing wrinkles rather than creating new ones. The predicted level of torque and shear force is slightly smaller than the bending rigidity, which aligns with AFM observations that OAM primarily increases the amplitude of existing wrinkles rather than generating many new ones.

The observation of wrinkle enhancement across the entire field of view, despite the expectation that shear force should be zero at the beam center, can be understood through the mechanics of the material response. The optical vortex induces a shear force that is indeed nonuniform and is zero at the beam center (the singular point of the vortex). However, the monolayer in this region is physically connected to the surrounding areas that do experience shear forces. This interaction can be analogized to applying a shear force to a sheet of paper by pulling its edges in opposite directions. Even though no direct force is applied to the center of the sheet, rumpling and deformations are still observed in the center due to the transmission of forces through the material. In our experiments, the position of the vortex center (singular point) is at the central point of the beam, where the intensity of the optical field is zero. Despite this, the mechanical coupling within the monolayer leads to the observed wrinkle enhancement across the entire field of view.

### Spin and Orbital Effects

**Figure 6:**
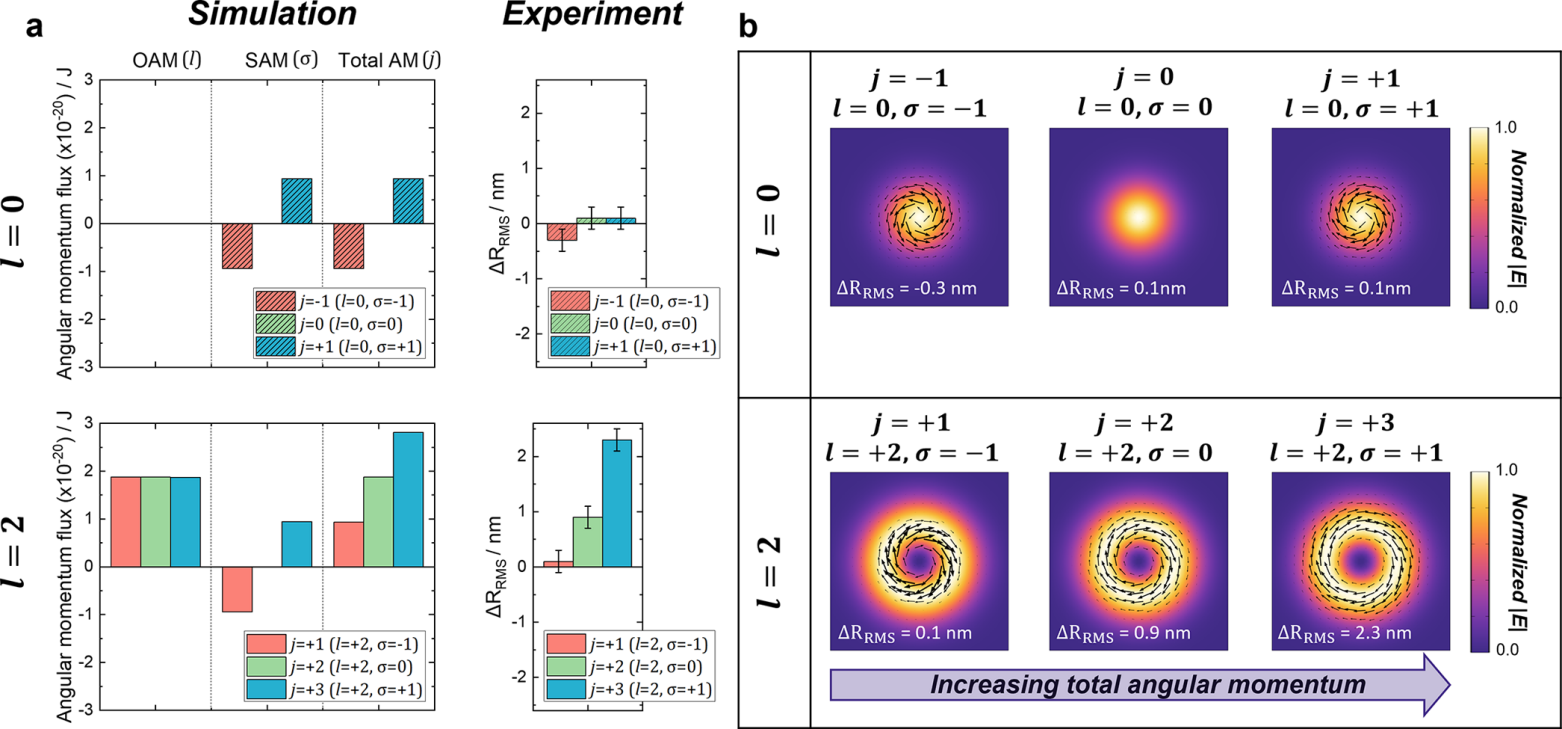
Numerical simulations and experimental data for monolayer graphene after illumination with beams possessing some combination of OAM and SAM. (a) Simulated angular momentum fluxes (OAM, SAM, total AM) for *l* = 0 beams (top row) and *l* = 2 beams (bottom row) combined with SAM, compared to the experimental change in root-mean-square roughness (ΔR_RMS_) obtained from a 10 × 10 μm AFM scan. (b) Simulated in-plane (transverse) momentum flux density for *l* = 0 (top row) and *l* = 2 (bottom row) beams combined with SAM, superimposed on the normalized electric field intensity. Arrows indicating linear momentum flux density are plotted on the same scale.

To gain deeper insights into the phenomena, we investigated beams with both orbital angular momentum (OAM, *l* = +2) and spin angular momentum (SAM, σ = −1, 0, + 1), resulting in total angular momentum *j* = *l* + σ values of +1 (*l* = +2, σ = −1), + 2 (*l* = +2, σ = 0), and +3 (*l* = +2, σ = +1), Figure 6. Additionally, we studied circularly polarized Gaussian beams (*l* = 0) with only spin angular momentum (σ = ± 1, *j* = ± 1). We assessed the relative ability of these beams to induce morphological changes using AFM and CVD monolayer graphene. The data clearly demonstrate a correlation between the change in the *R*_RMS_ caused by illumination and the *j* value of the beam (Figure 6a). In particular, we found that the beams with *j* = ± 1 did not produce a measurable increase in surface roughness, and note that this was true regardless of whether the total *j* = +1 resulted from a Gaussian beam with *l* = 0, σ = +1, or an LG beam with *l* = +2, σ = −1. This suggests that the magnitude of the deformations was associated with the total angular momentum content of the beam.

Using the same methodology as for Figure 5a,b, we performed numerical simulations of the in-plane momentum flux density for these beams. We see, in Figure 6b, that this quantity has an azimuthal component whenever the incident beam has a net angular momentum, be it orbital, spin, or a combination of the two. It is this azimuthal momentum flux that corresponds to the torque exerted.

In summary, the data indicate that the extent to which the material’s inherent wrinkles are amplified is correlated with the total angular momentum of the chiral light beams. Consideration of the in-plane momentum fluxes supports the interpretation that beams with higher total angular momentum apply correspondingly greater torques to the surface.

## Conclusions

Our work demonstrates an approach to manipulating electrical properties in 2D materials: optical strain engineering driven by the inherent sense of twist associated with the angular momenta possessed by chiral light beams. We achieve this by applying optical torques generated by Laguerre-Gaussian (LG) beams carrying OAM. Gaussian beams which do not carry angular momenta can only apply an out-of-plane force through the exchange of linear momentum, and are significantly less effective at inducing wrinkling.

While manipulating matter on mesoscopic scales using optical forces and torques (optomechanics) is well established,^12−14,47^ these forces were previously thought to be too weak for inducing significant strain and modifying electronic properties. This perception stemmed from the behavior of bulk materials requiring high pressures due to their strong atomic bonds. The inherent flexibility of 2D materials allows them to easily deform out-of-plane when subjected to in-plane shearing forces. This characteristic, combined with their low bending stiffness,^45,46^ makes them highly susceptible to strain induced by optical forces. Here, we highlight the critical role of angular momenta possessed by chiral light beams. We demonstrate that even weak optical forces can significantly impact 2D materials because beams carrying angular momentum can induce both normal and in-plane (shear) forces simultaneously. Essentially, the “push and twist” action of beams with angular momentum is more effective at rumpling and inducing strain compared to the “push” forces of achiral Gaussian beams. This approach also allows for the design of other light beam profiles (*e.g*., Hermite-Gaussian modes) to tailor the specific surface forces applied to the material. Advancements in vector light field control further facilitate the practical application of this method. These advancements enable precise design of optical profiles with specific polarization and phase vortices, allowing for the introduction of torques with desired strength, location and timing. This precise control offers significant advantages: increased flexibility and convenience in material manipulation, and dynamic control for both spatial (photopatterning) and temporal manipulation of strain, leading to reconfigurable material functionality.

The ability of chiral light beams to manipulate the band structure of monolayers of transition metal dichalcogenides (TMDCs), by tens of meV as suggested by luminescence, suggests potential applications for this effect. TMDCs are promising materials for next-generation electronics, where they could replace silicon as the semiconductor element in metal-oxide-semiconductor field-effect transistors (MOSFETs). The threshold voltage of a FET, which is the gate voltage required to create a conductive channel, is influenced by the band gap. Materials with larger/smaller band gaps generally have higher/lower threshold voltages. This reported phenomenon would enable optical control of the gate voltage and, consequently, the state of the transistor, leading to the development of a chiral light-based optical transistor.

Looking ahead, the control afforded by OAM beams may extend to manipulating the relative rotation of layers in 2D bilayers, a topic of growing interest due to their tunable band structures.^48,49^ Overall, optical strain engineering with chiral light beams is a paradigm for “writing” and “erasing” electronic properties of 2D materials for next-generation optoelectronic technologies.

## Methods

### Conductance Measurements from Monolayer Graphene

I–V curves were performed using a commercially available graphene field-effect transistor (GFET, Graphenea),^50,51^ which can be directly incorporated into the Graphenea card (Figure S1). The monolayer graphene is grown by CVD and transferred onto a 90 nm SiO_2_/Si substrate. A DC power supply (Rapid HY3005–3) was used to apply a voltage between the source and the drain (*V*_ds_). The drain-source current (*I*_ds_) was measured using a multimeter (True RMS digital multimeter DM-441B). The maximum drain-source voltage used for the measurements was 0.6 V to avoid heating in the GFET.

For laser illumination of the GFET, an optical set up with a collimated 405 nm laser was used (Figure S2). A λ/2 plate and a polarizer were used to control the excitation power and the beam is linearly polarized. In this study we use a spiral phase plate^52,53^ (SPP) to generate the LG beam (*l* = 2, Vortex photonics, 405 nm), although other recent methods have been developed.^54−58^ The SPP is placed along the optical axis to generate the LG beam, and it is placed in an *xy* mount to allow proper alignment of the center of the spiral phase plate with respect to the optical axis. The beam was directed toward the sample using a 50:50 beam splitter and a 10× objective (NA = 0.25). The excitation power was measured between the beam splitter and the objective lens. A 10:90 beam splitter was used to direct 10% of the light toward a camera, where the sample can be visualized.

In Figure 2c,d, each measurement with *l* = 0 and *l* = 2 beams was performed on a pristine monolayer graphene *i*.*e*. a fresh monolayer that had never been illuminated with the laser beam. The error bars in Figure 2d were obtained from the standard error of linear fits of the *I*–*V* characteristics.

### Raman Mapping of Monolayer Graphene

Raman mapping of the GFET (Graphenea) was done using LabRAM HR Evolution, using a laser excitation wavelength of 532 nm (excitation power: 500 μW) and 50X objective (NA = 0.5). LG beam illumination of the GFET was done using the same optical set up of Figure S2, equipped with a 405 nm laser (excitation power: 500 μW), 10X objective (NA = 0.25). After LG beam illumination, the Raman mapping was conducted immediately after, and the mapping took an overall time of 2 hours to be completed.

### AFM Measurements of Graphene

Atomic force microscopy (AFM) measurements were performed using a Dimension Icon Atomic Force Microscope System with ScanAsyst with a silicon tip (ScanAsyst-Air-HPI). AFM was performed on a commercially available (1 cm × 1 cm) monolayer graphene sample grown by CVD and transferred onto 90 nm SiO_2_/Si substrate (Graphenea). A scan size of 10 μm, a scan rate of 0.3 Hz and 512 samples/line were used for all the scans collected. The variations in roughness and waviness of the surface across the white cut lines of Figure 3d,e were plotted using Gwyddion software. Roughness and waviness represent high-frequency and low-frequency profile along the surface, respectively. In Figure 3g,h it is assumed that the average of the mean value of the roughness is 0 (*r*_*j*_ = *z*_*j*_ – *z̅*, where *r*_*j*_ is the roughness at the *j*th position, *z*_*j*_ is the height of the position, and *z̅* is the mean of all *z*_*j*_).

For LG (*l* = 2) and Gaussian beam illumination, an excitation power of 500 μW was used.

The error bars in the AFM data presented in Figure 6a were obtained from the standard errors between the change in root-mean-square roughness (Δ*R*_RMS_) of 2 different AFM scans taken at the same spot with a different AFM tip.

### Numerical Simulations of Laguerre-Gaussian Beams and Laser Heating

Laguerre-Gaussian beams were simulated in COMSOL Multiphysics (version 6.0) using the electromagnetic wave frequency domain module and scattering boundary conditions. The beam was modeled using the equation for a Laguerre-Gaussian beam, which in cylindrical coordinates takes the form^59^
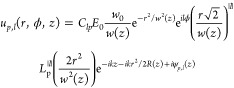
1awhere *p* is the radial mode (in our case *p* = 0) and *l* the topological charge. The other parameters are defined as^59^
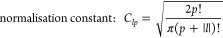
2
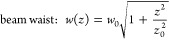
3
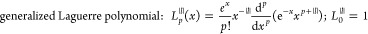
4
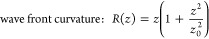
5
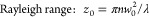
6
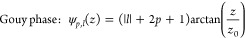
7As expected, the intensity ring in the LG beam increases in size for increasing *l*, and the number of helices in the phase front is equal to *l*. Reversing the sign of *l* reverses the rotational sense of the phase fronts (Figure S7).

To further test the validity of the model, the angular momentum (AM) flux was simulated in air as derived by Barnett^60^
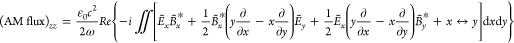
8As expected for a transverse-normalized Laguerre-Gaussian beam, the AM flux was found to follow a linear relationship for increasing topological charge, which agrees with the fact that orbital angular momentum per photon increases linearly as ± *l*ℏ (Figure S8).

To simulate monolayer graphene and WS_2_, a 2 nm thick layer was used due to constraints in the minimum element size in the mesh. To match the experimental beam waists, the simulations involving graphene and WS_2_ were done with a beam waist of 12.4λ and 2λ, respectively. Real and imaginary refractive indices for monolayer graphene and monolayer WS_2_ were taken from Weber et al.^61^ and Jung et al.,^62^ respectively.

To simulate the laser heating of Gaussian and Laguerre-Gaussian beams, the heat transfer module was used, coupled to the electromagnetic wave frequency domain via electromagnetic heating. In the heat transfer module, a temperature boundary condition was used at the input (air) surface with a fixed temperature of 293.15 K. The electromagnetic power loss density was used as a heat source, which results from coupling the electromagnetic waves and heat transfer modules.

### PL Spectroscopy of Monolayer WS_2_

The exfoliated monolayer WS_2_ flakes on SiO_2_/Si substrate were obtained from 2D semiconductors. The CVD-grown monolayer WS_2_ (1 cm × 1 cm) on a sapphire substrate was obtained from Ossila.

The PL spectroscopy of WS_2_ was performed using a home-built optical set up (Figure S10). The optical set up includes a collimated 532 nm laser diode as the excitation source, and an excitation power of 100 μW was used. A spiral phase plate (SPP) was then placed along the optical axis to generate the LG beam (*l* = 4, Vortex photonics). As before, the SPP was placed in an *xy* mount to allow proper alignment of the center of the spiral phase plate with respect to the optical axis. The beam was directed toward the sample using a 50:50 beam splitter and a 60X objective (NA = 0.9). PL is collected in a reflection geometry through the same objective and a 10:90 beam splitter was used to direct 10% of the light toward a camera, where the sample can be visualized, and 90% of the signal toward an optical fiber, which carries the photoluminescence signal to the CCD camera (Newton CCD, Andor Technology).

PL spectra were fitted using Gaussian fits and the error bars of Figures 4, S11, and S12 were obtained from the standard error of the fittings.

## Data Availability

All data are available in the manuscript and the Supporting Information.

## Supporting Information Available

Additional OAM transfer experiments and data using Raman spectroscopy and AFM of graphene and photoluminescence of monolayer WS_2_. Data regarding experimental heating and laser power dependency of monolayer WS_2_ are also included. Furthermore, the file contains numerical simulations of laser heating for both monolayer graphene and WS_2_ using Laguerre-Gaussian beams. Additionally, the theory developed for the momentum and angular momentum flux densities inside a medium is provided (PDF)

## Pre-print manuscript

A preprint version of this manuscript was previously published, prior to the publication of this current manuscript: P.L.L.; P.S.; N.M.; F.C.; R.K.; S.K.C.; A.S.; A.M.; A.G.; D.M.; S.F.; J.B.G.; S.M.B.; N.G.; M.K., Optical Strain Engineering Enables Dynamic Spatial Control of 2D Nanomaterial Properties. 2023. *Research Square*. https://www.researchsquare.com/article/rs-3499143/v1 (accessed: October 30, 2023).
