# Supplementary material for: Spatial Control of 2D Nanomaterial Electronic Properties Using Chiral Light Beams
Source: ACS Nano. 2024 Jul 29;18(31):20401–11. doi: 10.1021/acsnano.4c04506 (PMC11313125; doi:10.1021/acsnano.4c04506)
Supplement: Supplementary file 1 — nn4c04506_si_001.pdf [file nn4c04506_si_001.pdf]

Supplementary information for
Spatial Control of 2D Nanomaterial Electronic Properties using Chiral Light Beams

Paula L. Lalaguna[1*], Paul Souchu[1,2], Neel Mackinnon[3], Frances Crimin[3], Rahul Kumar[1], Shailendra Kumar Chaubey[1], Asma Sarguroh[1], Amy McWilliam[3], Alexey Y. Ganin[1], Donald A. MacLaren[3], Sonja Franke-Arnold[3], Jörg B. Götte[3], Stephen M. Barnett[3], Nikolaj Gadegaard[4] & Malcolm Kadodwala[1*]

[1] School of Chemistry, University of Glasgow, Glasgow G12 8QQ, UK.

[2] Faculté des sciences et ingénierie, Université de Toulouse UPS, Toulouse 31400, France

[3] SUPA, School of Physics and Astronomy, University of Glasgow, Glasgow G12 8QQ, UK.

[4] James Watt School of Engineering, University of Glasgow, Glasgow G12 8QQ, UK.

*Email to: p.laborda-lalaguna.1@research.gla.ac.uk, malcolm.kadodwala@glasgow.ac.uk

Table of Contents

1. Set up for I-V measurements of monolayer graphene 2
2. Beam profile of 405nm laser 3
3. Raman spectroscopy of monolayer graphene 3
4. AFM of graphene when illuminated with a Gaussian beam 5
5. Finite-element electromagnetic simulations of Laguerre-Gaussian beams in air 6
6. Finite-element simulated laser heating of Laguerre-Gaussian beams in monolayer graphene on SiO_2/Si 7
7. Photoluminescence spectroscopy set up 8
8. Photoluminescence spectroscopy of monolayer WS_2 9
9. Response of monolayer WS_2 to temperature 11
10. Finite-element simulated laser heating of Laguerre-Gaussian beams in monolayer $WS_2$12
11. Power-dependent photoluminescence on CVD-grown and exfoliated monolayer WS_2 .13
12. Derivation of momentum flux density inside a medium 14
13. Derivation of angular momentum continuity equation inside a medium 17
14. Calculation of net forces and torques imparted on a material....................19
15. Supplementary numerical simulations of angular momentum flux in WS_2....................22

1. Set up for I-V measurements of monolayer graphene

An image of the Graphenea card where the GFET (graphene field effect transistor) is incorporated into is shown in Figure S1a. This includes switches for the drain and the source, and individual sources for each FET device. Each graphene channel can be selected with the external individual source switches. A schematic of the electric circuit is shown in Figure S1b.

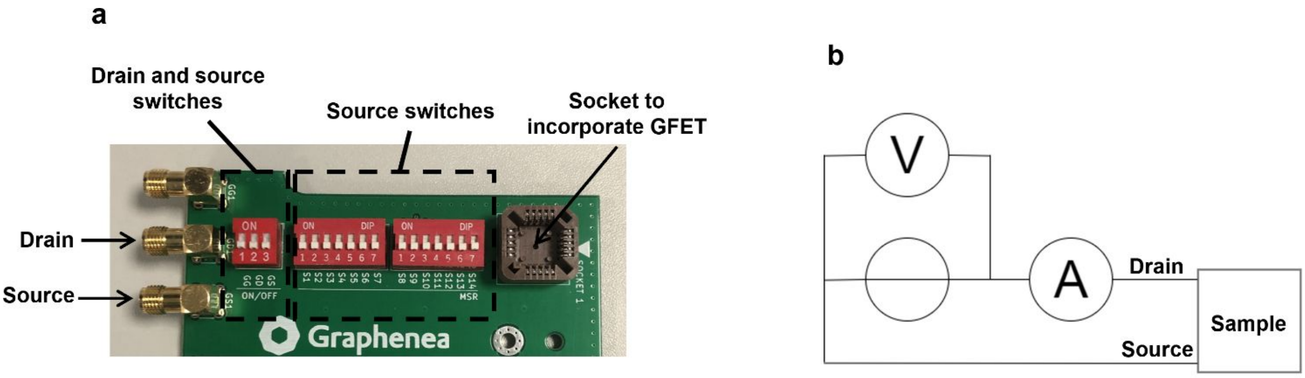

Figure S1. (a) Image of the card (Graphenea) where the GFET can be incorporated. (b) Electrical circuit of the GFET.

A schematic of the optical set up for laser illumination of the monolayer graphene is displayed in Figure S2 (see Methods for full description).

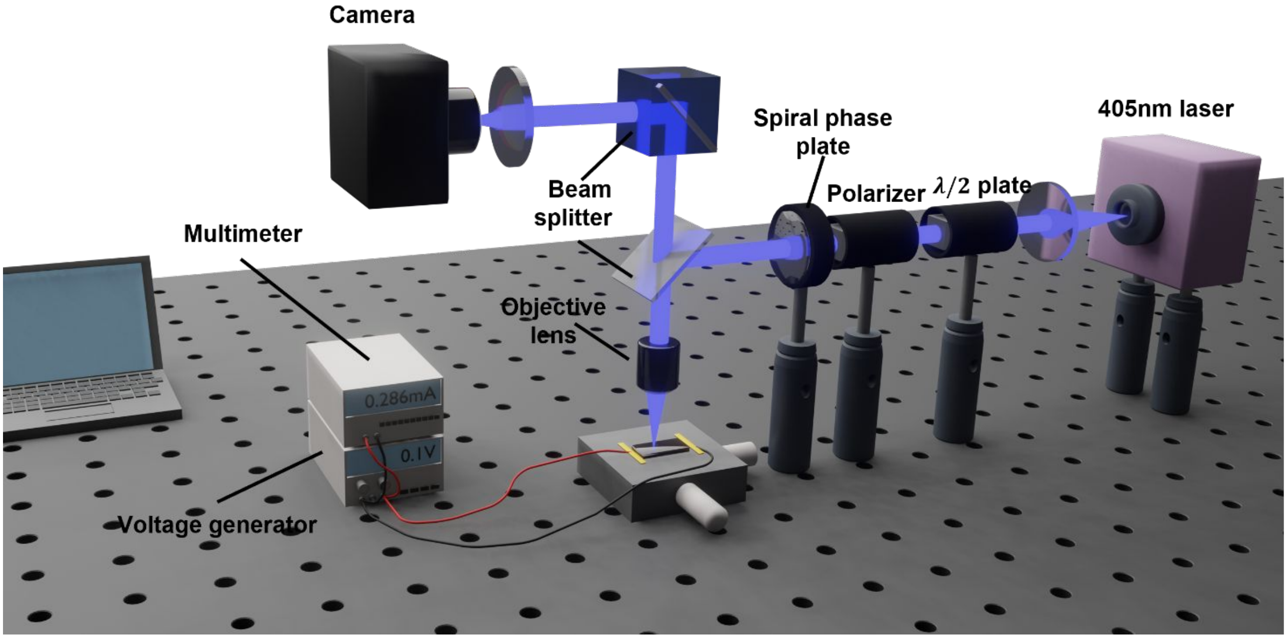

Figure S2. Schematic of the optical set up used to illuminate the GFET with Gaussian and LG beams and conduct I-V curves. The LG beam is generated by inserting a spiral phase plate ($l = 2$, 405 nm, Vortex Photonics) along the optical path. The beam is directed towards the sample with a 50:50 beam splitter and a 10X objective (NA=0.25).

2. Beam profile of 405nm laser

Measured beam profiles for l = 0 and l = 2 beams are depicted in Figure S3a. The profiles were fitted to the intensity function $I(r)$ corresponding to the electric field of the LG beam squared. Recall that $w(z)$ = w_0 at the focal point z = 0, where w_0 is the beam waist, so the intensity function becomes:

$$I(r) = |E(r)|^2 = Ae^{\frac{-2(r-r_0)^2}{w_0^2}} \left(\frac{(r-r_0)\sqrt{2}}{w_0} \right)^{2|l|}$$

A is the peak height, r is the beam radius and r_0 is the profile centre. l is the topological charge, so for l = 0 the intensity function reduces to that of a Gaussian beam. The fitted data and extracted beam waists and diameters for l = 0 and l = 2 beams are given in FigureS3b and c, respectively. For an LG beam with $l \neq 0$ the beam diameter may be defined as the separation between intensity maxima,[1] though a more convenient measure may be the root mean squared intensity, as this definition may be applied also to the $l = 0$ Gaussian beam.[2] For an LG beam with radial index $p = 0$ and azimuthal index l, the latter definition gives a diameter of $d = w_0\sqrt{2(|l|+1)}$. The $l = 0$ and $l = 2$ beams give beam diameters of 20.6 and 32.6 µm, respectively. For comparison, the size of the AFM images in Figures 3 and S6 is 10 µm x10 µm.

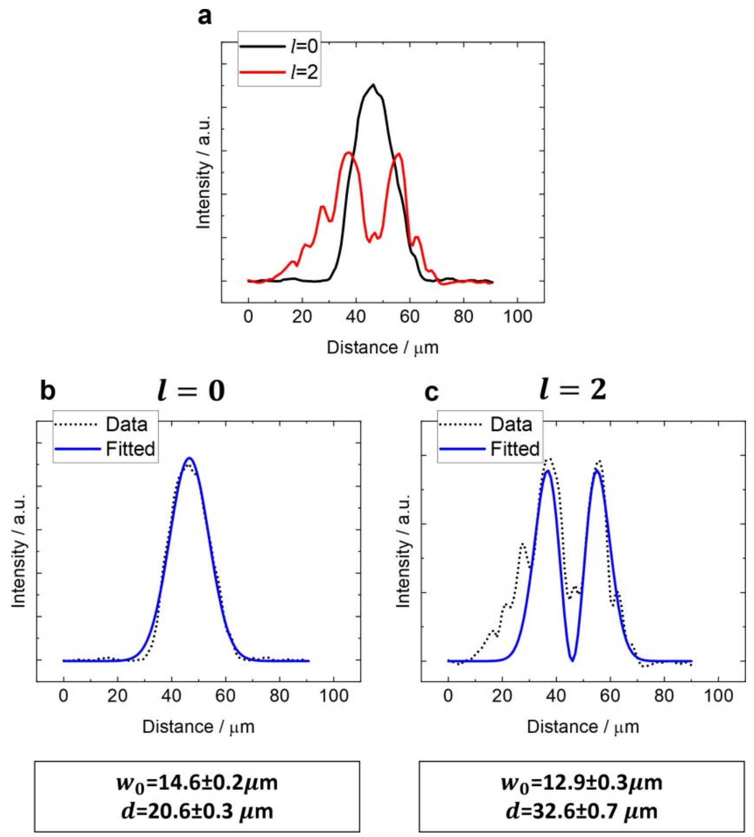

Figure S3. (a) Measured beam profiles for $l = 0$ (black) and $l = 2$ beams (red) used to conduct conductance measurements, Raman spectroscopy and AFM of monolayer graphene. (b) Experimental Gaussian beam profile (black) fitted to the intensity expression with $l = 0$ (blue). (c) Experimental LG beam profile (black) fitted to the intensity expression with $l = 2$ (blue). For (b) and (c), the extracted beam waist w_0 and beam diameter d are indicated underneath.

3. Raman spectroscopy of monolayer graphene

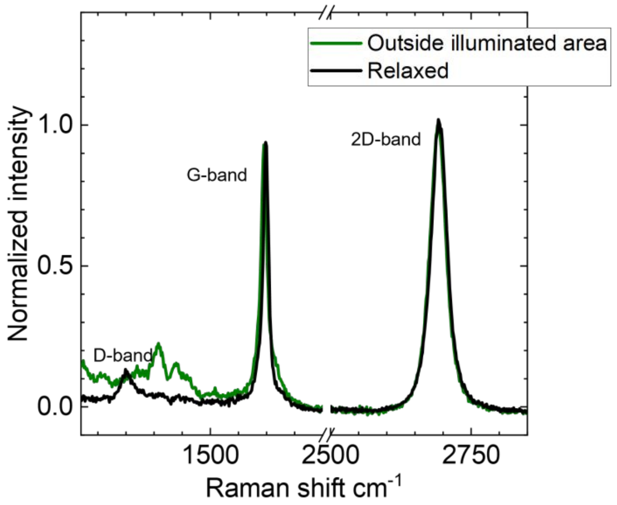

Figure S4. Raman spectra collected at the illuminated area with an LG beam ($l = 2$) after a relaxation period >48 hours (black) and outside illuminated area (green). The spectra have been normalised to the maximum intensity of the 2D-band.

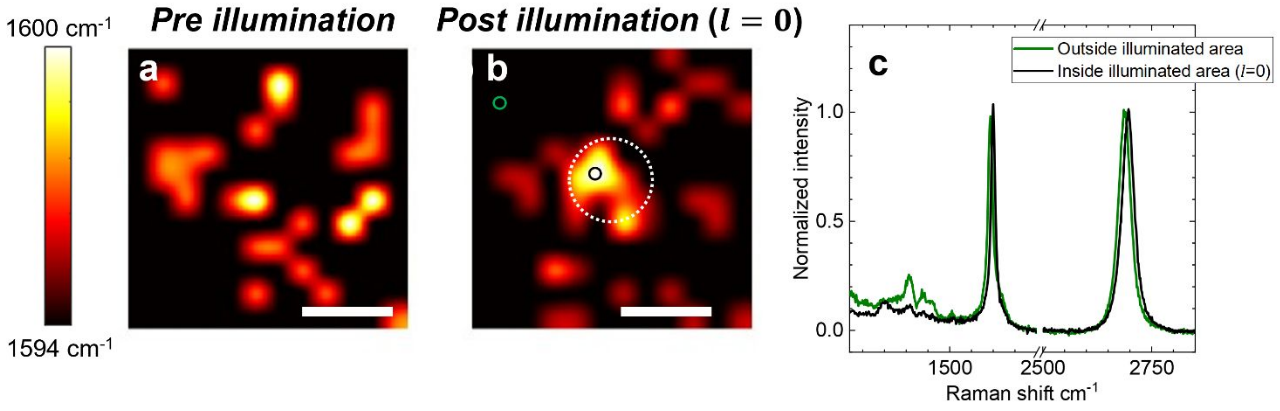

Figure S5. (a,b) Raman mapping of the G-band position before illumination (a) and after Gaussian beam ($l = 0$) illumination (b) (scale bar: 30μm). The approximate illuminated area in panel (b) is indicated with a white dotted circle. (c) Raman spectra for illuminated (black) and unilluminated (green) areas collected at the positions depicted by the black and green circles, respectively, in panel (b). The spectra have been normalised to the maximum intensity of the 2D-band.

4. AFM of graphene when illuminated with a Gaussian beam

Supplementary AFM measurements collected with a Gaussian beam ($l = 0$) are shown in Figure S6. Figure S6 shows that the changes in contrast after illuminating with Gaussian beam are much smaller than those observed with LG beam ($l = 2$) in Figure 3 of the main manuscript. Furthermore, the root-mean-square roughness (R_{RMS}) only increases by 0.3 nm.

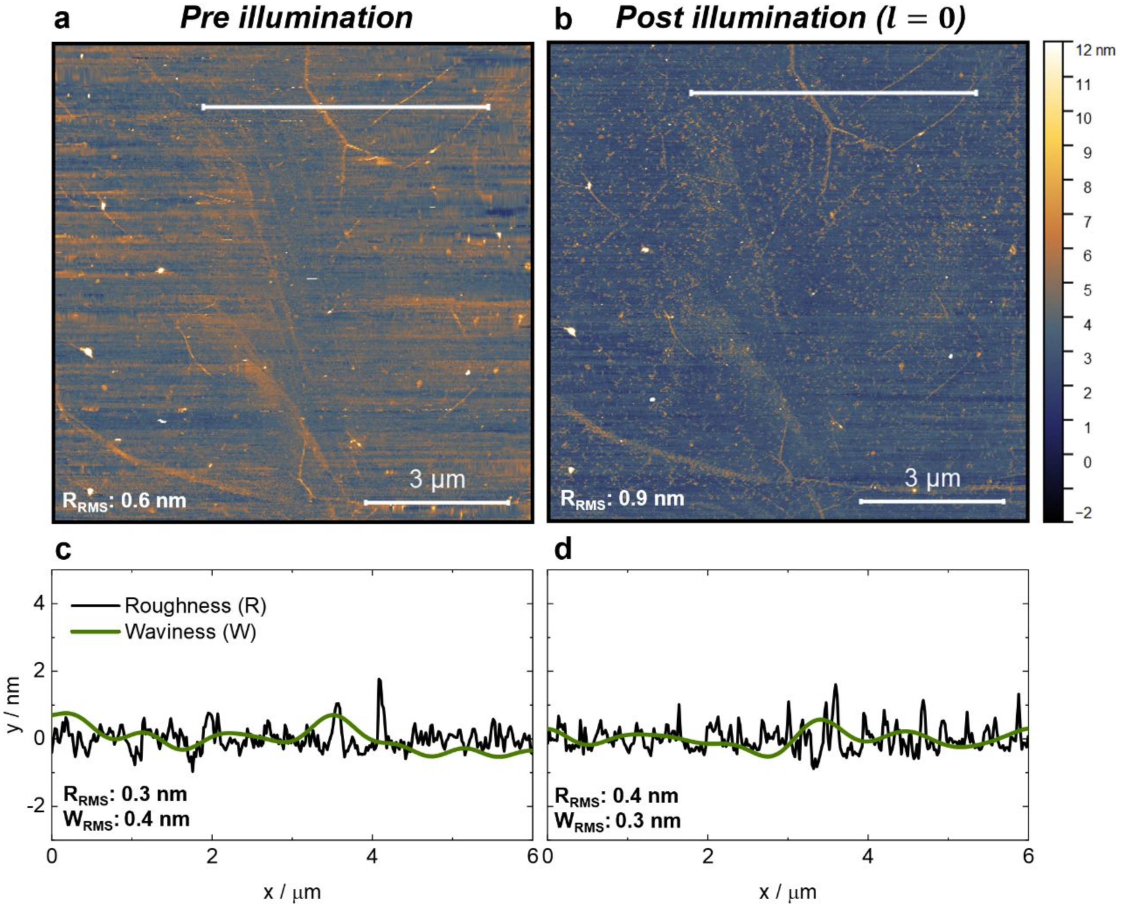

Figure S6. (a,b) AFM characterisation of graphene before illumination (a) and after illumination (b) with Gaussian ($l = 0$) beam (excitation power: 500 μW). The root-mean-square roughness (R_{RMS}) of both panels (a) and (b) is displayed at the bottom left corner. (c,d) Variation in roughness (black) and waviness (green) across the white cut lines in (a) and (b), respectively. The root-mean-square roughness (R_{RMS}) and waviness (W_{RMS}) have been added at the bottom left corner. See Methods for details.

5. Finite-element electromagnetic simulations of Laguerre-Gaussian beams in air

Electromagnetic simulations of Laguerre-Gaussian beams (see Methods for details) were first tested in air. Figure S7 shows that the size of the LG beam increases for larger topological charge as expected, and the rotational sense of the phase fronts is reversed upon changing the sign of the topological charge.

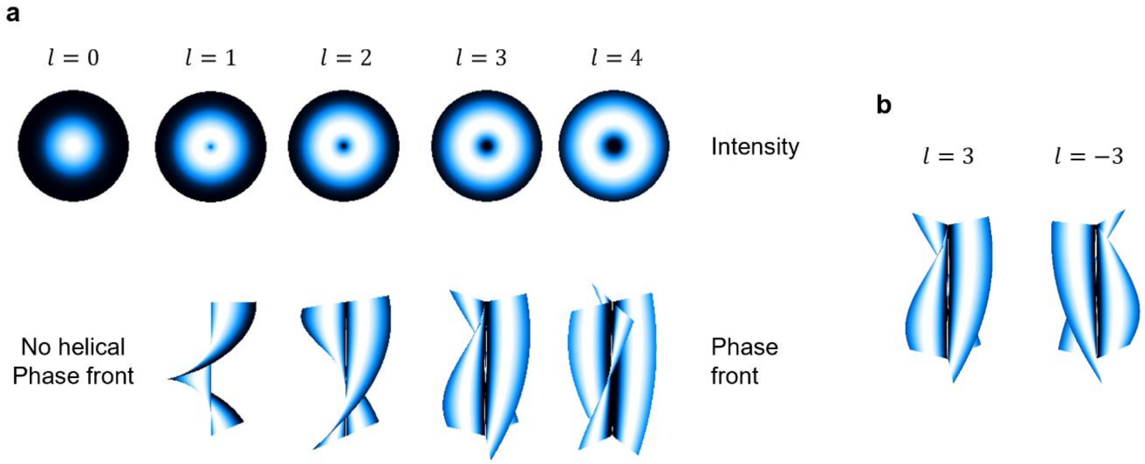

Figure S7. (a) Simulated intensity and phase front of LG beams in air for varying topological charges. (b) Phase fronts for $l = 3$ and $l = -3$ beams.

The validity of the simulations was also checked by simulating the angular momentum (AM) flux[3] in air. The results from these simulations show that the AM flux increases linearly as a function of topological charge (Figure S8a) and power (Figure S8b) as expected.

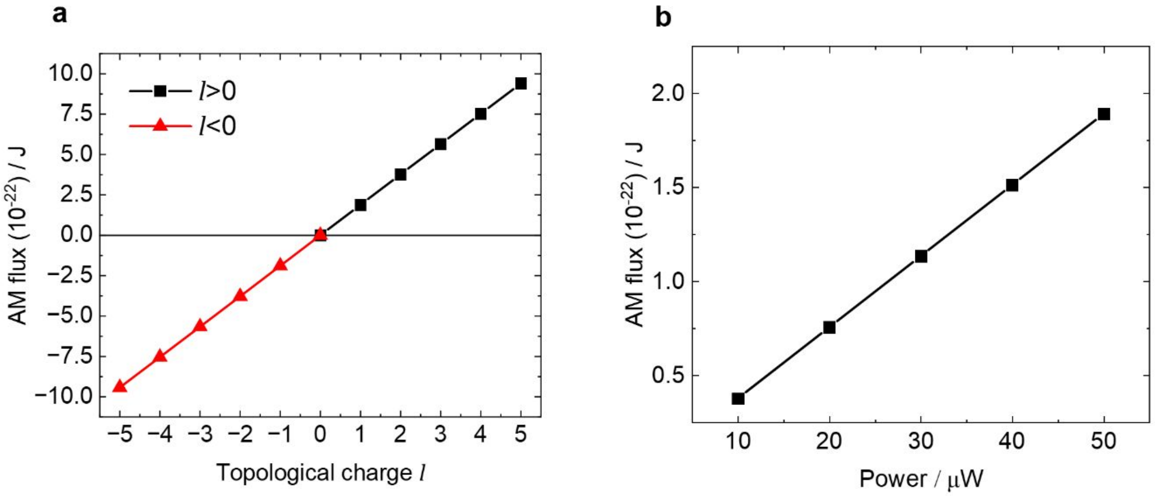

Figure S8. (a) Angular momentum (AM) flux in air as a function of topological charge for a transverse-normalised Laguerre-Gaussian beam. Excitation power: $50\mu W$. (b) AM flux in air as a function of excitation power.

6. Finite-element simulated laser heating of Laguerre-Gaussian beams in monolayer graphene on SiO_2/Si

Laser heating simulations (see Methods for details) were performed for monolayer graphene on SiO_2/Si, and the results are shown in Figure S9. It is obtained that LG beams for increasing topological charges produce smaller temperature rises than a Gaussian beam, as the power is distributed over a larger area.

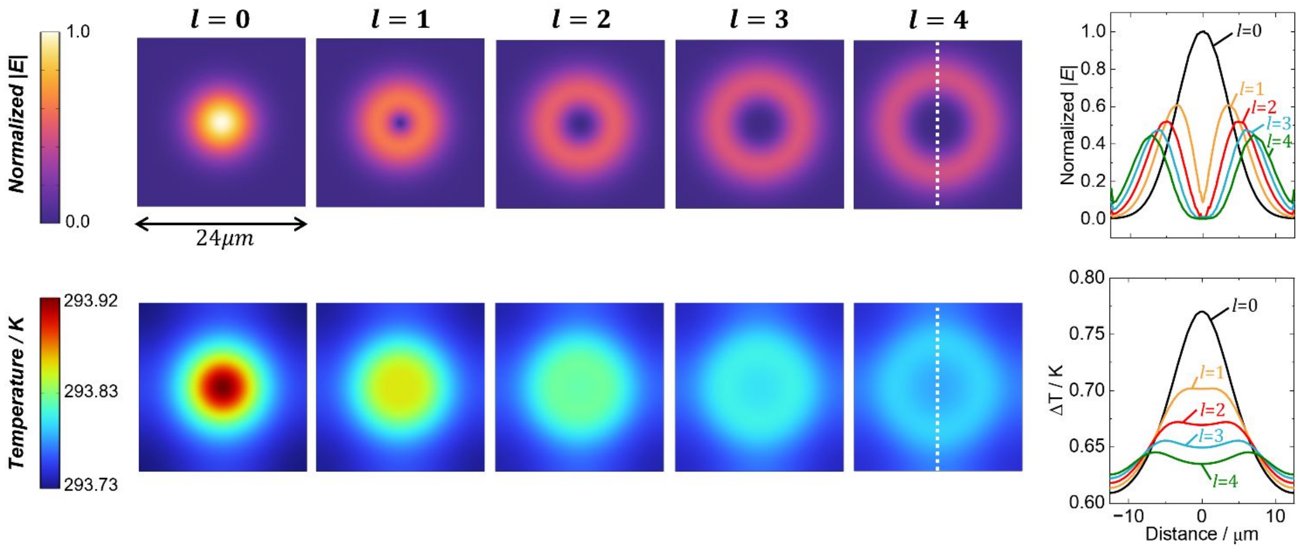

Figure S9. Simulated electric field intensity and temperature at graphene-substrate interface ($w_0 = 12.4\lambda$, power: $1mW$) for Gaussian beam and Laguerre-Gaussian beams of varying topological charges in monolayer graphene on SiO_2/Si. A cut line (white line) of the electric field intensity and temperature rise with respect to the initial temperature $T_0 = 293.15\ K$ is displayed on the right-hand side.

7. Photoluminescence spectroscopy set up

A schematic of the photoluminescence (PL) set up used to conduct PL spectroscopy of monolayer WS_2 is shown in Figure S10 (see Methods for full description).

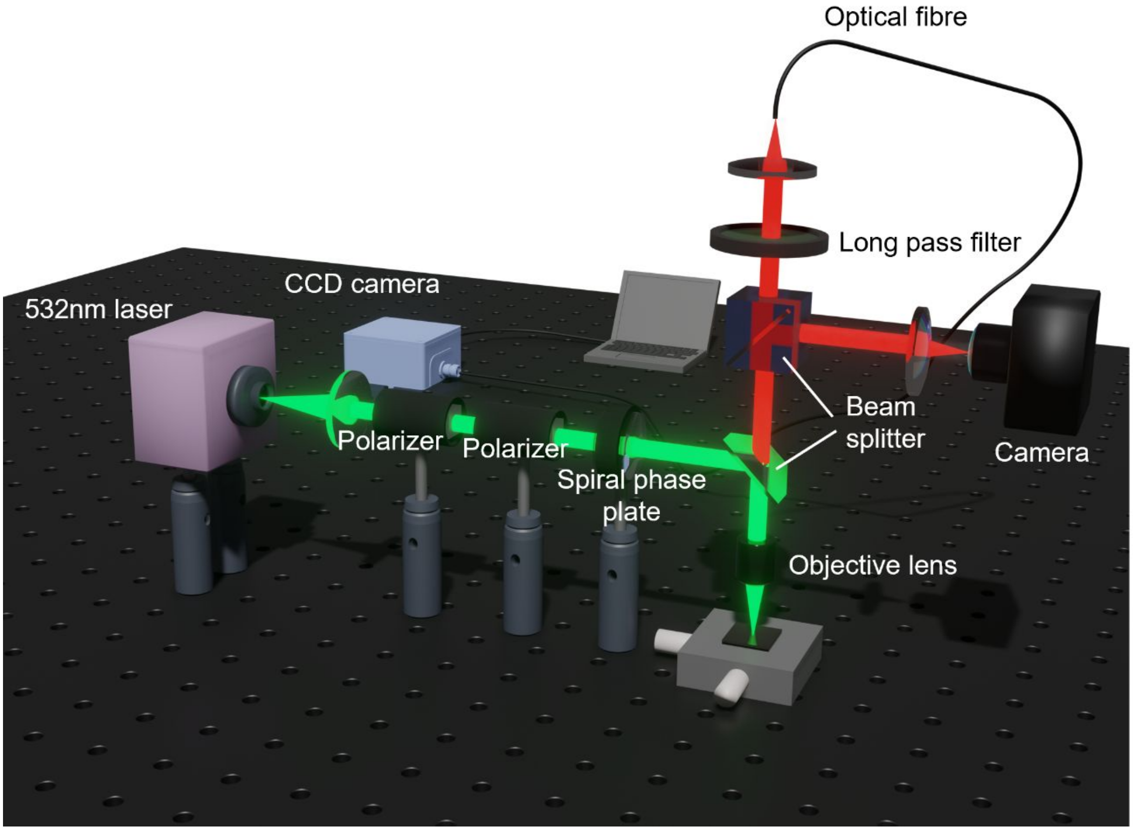

Figure S10. Schematic of the PL set up which includes a 532nm laser and the resulting red photoluminescence from monolayer WS_2. The LG beam is generated by inserting a spiral phase plate ($l = 4$, 532 nm, Vortex photonics) along the optical path. The beam is directed towards the sample with a 50:50 beam splitter and a 60X objective (NA=0.9).

8. Photoluminescence spectroscopy of monolayer WS_2

The changes observed for exfoliated monolayer with LG beams are displayed in Figure S11.

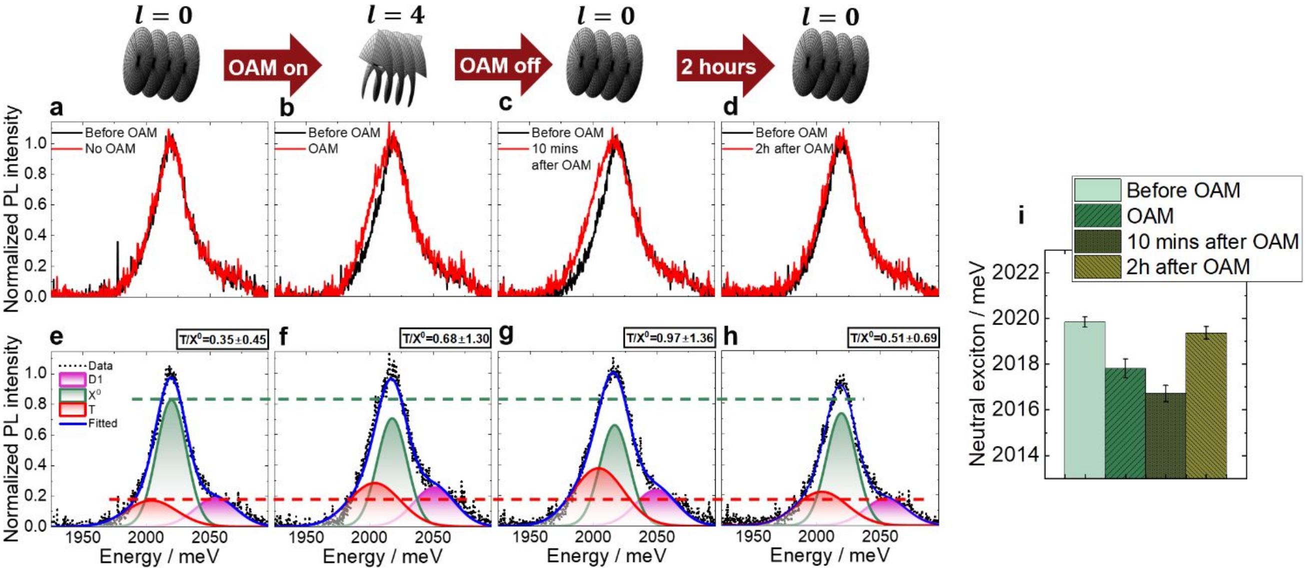

Figure S11. (a) Reference spectra with no OAM (red) and right before OAM is introduced (black), showing no change between the two consecutive spectra. (b) Spectrum collected with OAM $l = 4$ (red) compared to the spectrum before OAM (black). (c,d) Spectrum collected with a Gaussian beam (c) 10 minutes and (d) 2 hours after OAM (red), compared to the spectrum before OAM (black). (e,f,g,h) Gaussian fits for the red spectra shown in panels (a,b,c,d) respectively. The green and red horizontal lines indicate changes in neutral exciton (X^0) and trion (T) emissions, respectively, and the T/X^0 intensity ratio is indicated at the top right corner. (i) Changes in the energies of the neutral exciton (X^0) obtained from the fittings shown in panels (e,f,g,h).

The red shift in PL observed with LG beams across different positions in the CVD-grown monolayer and exfoliated monolayer flakes is shown in Figure S12a and Figure S12b, respectively.

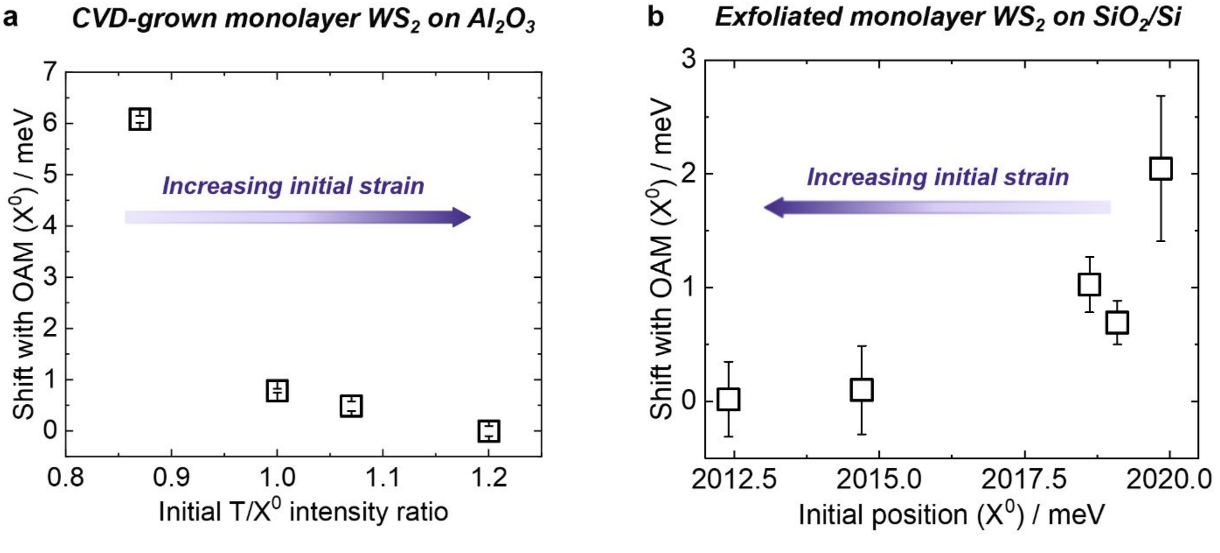

Figure S12. (a) Shift with OAM for the neutral exciton (meV) as a function of T/X^0 intensity ratio in the CVD-grown monolayer. (b) Shift with OAM for the neutral exciton (meV) as a function of the initial position of the neutral exciton (meV) in exfoliated monolayer sample. The error bars were obtained from Gaussian fittings of experimental data.

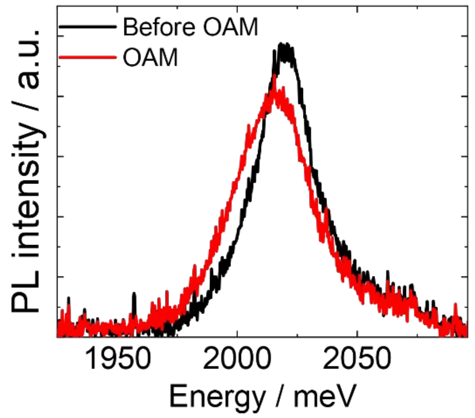

Figure S13. Unnormalized PL spectra of an exfoliated monolayer WS_2 flake before and after OAM illumination.

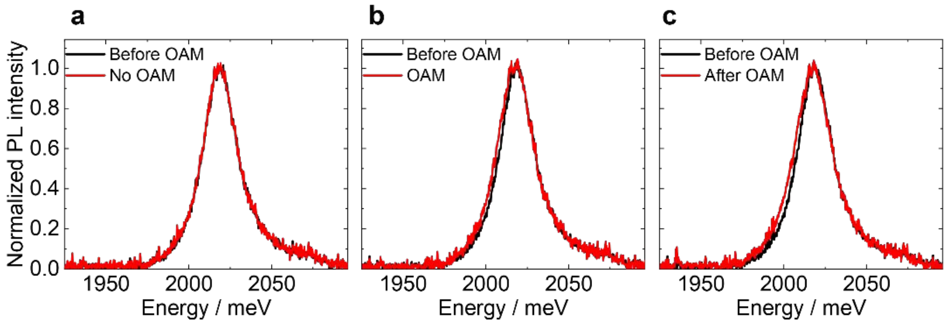

Figure S14. OAM-induced red shift in PL collected from an exfoliated monolayer flake with the opposite sign of OAM ($l = -4$) to that shown in the main text (see Figure 4). (a) Reference spectra with no OAM (red) and right before OAM is introduced (black), showing no change between the two consecutive spectra. (b) Spectrum collected with OAM $l = -4$ (red) compared to the spectrum before OAM (black). (c) Spectrum collected with a Gaussian beam after OAM (red), compared to the spectrum before OAM (black).

9. Response of monolayer WS_2 to temperature

Heating of monolayer WS_2 was carried out using a commercial heating table (Linkam Scientific CO 102). Heating was performed from 20 °C to 50 °C in increasing steps of 10 °C. Before collecting the PL spectrum, the sample was left to stabilise at that temperature for 1h.

The changes in the PL spectra associated with heating the CVD-grown monolayer WS_2 on Al_2O_3 are shown in Figure S15a, showing a red shift of the PL emission. The energies of the neutral exciton and trion decrease as a function of temperature at approximately the same rate (Figure S15b), contrary to our observations with OAM (Figure 4g,h in the main manuscript). Furthermore, the trion-to-neutral exciton intensity ratio only changes significantly for a temperature of 40 ℃ and above (Figure S15c).

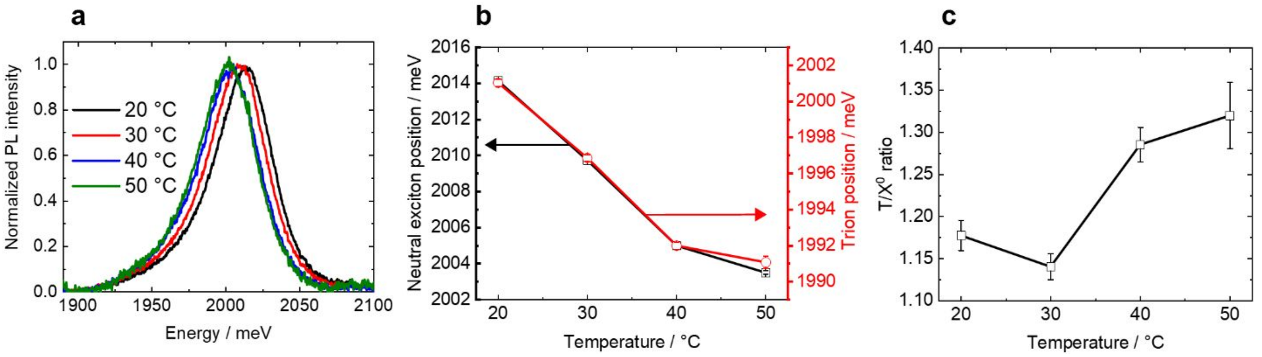

Figure S15. (a) Normalised PL spectrum of CVD-grown monolayer WS_2 on Al_2O_3 as a function of temperature. (b) Energy positions for neutral exciton energy (black) and trion energy (red) as a function of temperature. (c) Trion-to-neutral exciton ratio as a function of temperature. The energy positions and T/X^0 ratio were obtained from Gaussian fittings of the experimental data in (a).

Experimental heating of an exfoliated monolayer flake is shown in Figure S16, also showing that the energy of the neutral exciton and trion decrease at the same rate within fitting error, and the trion-to-neutral exciton ratio only changes significantly for a temperature of 40 ℃ and above.

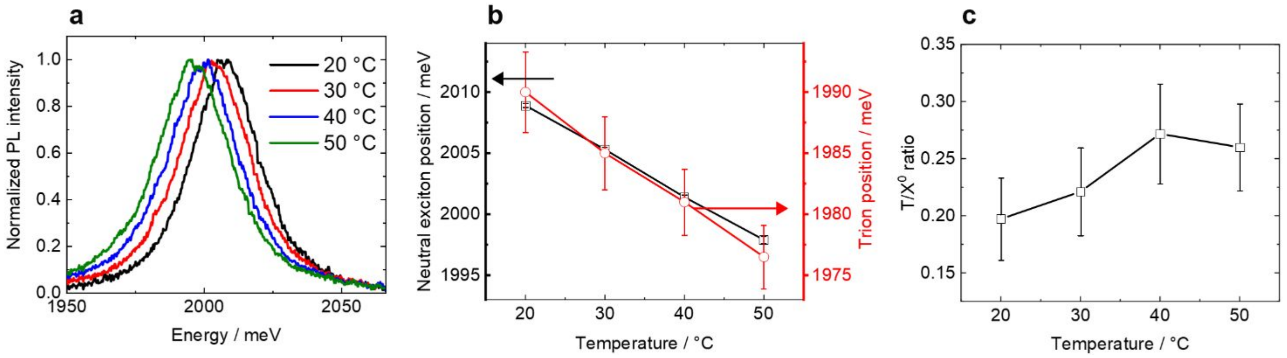

Figure S16. (a) Normalised PL spectrum of an exfoliated monolayer WS_2 flake on SiO_2/Si as a function of temperature. (b) Energy positions for neutral exciton energy (black) and trion energy (red) as a function of temperature. (c) Trion-to-neutral exciton ratio as a function of temperature. The energy position and T/X^0 ratio were obtained from Gaussian fittings of the experimental data in (a).

10. Finite-element simulated laser heating of Laguerre-Gaussian beams in monolayer WS_2

Laser heating simulations (see Methods for details) were performed for monolayer WS_2 on the two different substrates used, Al_2O_3 and SiO_2/Si, and the results are shown in Figure S17 and Figure S18, respectively. In both cases, it is obtained that LG beams for increasing topological charges produce smaller temperature rises than a Gaussian beam, as the power is distributed over a larger area.

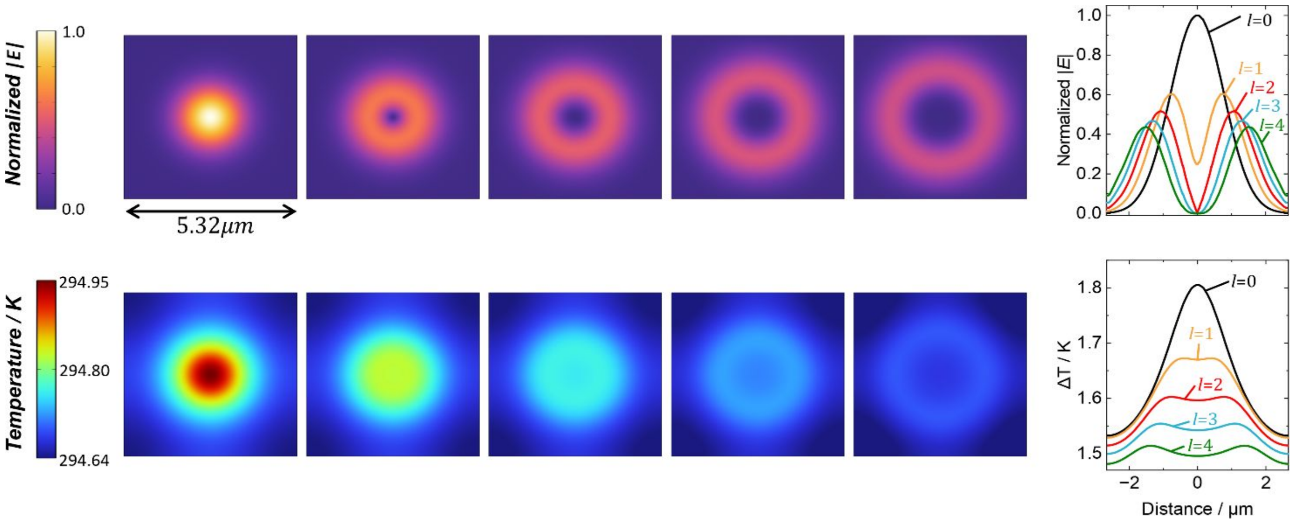

Figure S17. **Simulated electric field intensity and temperature for Gaussian and Laguerre-Gaussian beams of varying topological charges in monolayer WS_2 on Al_2O_3. Excitation power: $200\mu W$. A cut line (white line) of the electric field intensity and temperature rise with respect to the initial temperature $T_0 = 293.15\ K$ is displayed on the right-hand side.**

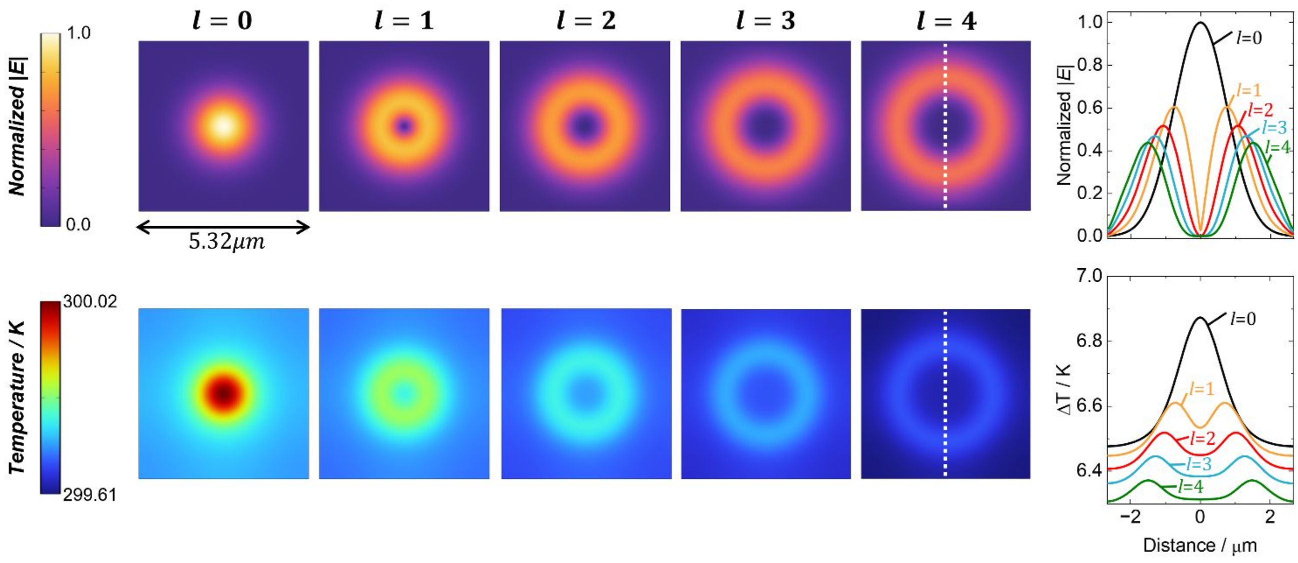

Figure S18. **Simulated electric field intensity and temperature for Gaussian and Laguerre-Gaussian beams of varying topological charges in monolayer WS_2 on SiO_2/Si. Excitation power: $200\mu W$. A cut line (white line) of the electric field intensity and temperature rise with respect to the initial temperature $T_0 = 293.15\ K$ is displayed on the right-hand side.**

11. Power-dependent photoluminescence on CVD-grown and exfoliated monolayer WS_2

The power-dependent PL spectrum of CVD-grown full-coverage monolayer WS_2 on Al_2O_3 is shown in Figure S19a, where the energy and trion-to-neutral exciton intensity ratio do not change with laser power, in agreement with the literature.[4] In exfoliated monolayer WS_2, the trion is particularly dominant at large excitation powers (Figure S19b), in agreement with the literature.[5] As LG beams have less power density than Gaussian beams (Supplementary section 9), the red shift and appearance of a shoulder at lower energy with LG beam illumination cannot be accounted for by a change in power density of the beam in the exfoliated monolayer, as reduced power density would result in a blue shift, not a red shift.

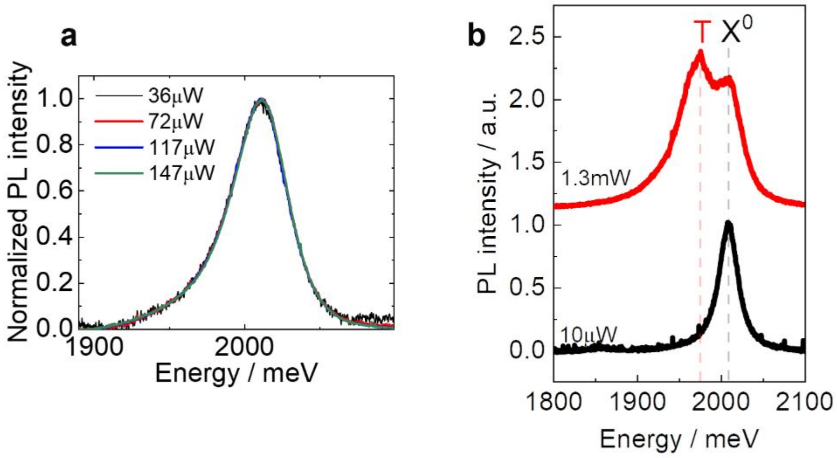

Figure S19. (a) Power-dependent PL in CVD-grown monolayer WS_2 on Al_2O_3. (b) Power-dependent PL in exfoliated monolayer WS_2 flakes on SiO_2/Si.

12. Derivation of momentum flux density inside a medium

In this section, we use the local continuity of momentum within a dielectric medium to derive an expression for the electromagnetic momentum flux tensor inside the medium. The conservation of the i-th component of electromagnetic momentum inside the medium can be expressed using the continuity equation:

$$\frac{\partial g_i}{\partial t} + f_i = -\nabla_j T_{ij}, \tag{1}$$

where g_i is the momentum density, f_i is the Lorentz force density, and T_{ij} is the momentum flux tensor (the ij-th component of T_{ij} is the flux of the i component of momentum in the j direction). A sum over repeated indices is implied. If we now write down expressions for the momentum density $\boldsymbol{g}$**,** and the Lorentz force density, $\boldsymbol{f}$, we can obtain an expression for T_{ij} from the requirement that equation (1) is satisfied.

In free space, the momentum density of light is straightforwardly given by $\mathbf{g} = \frac{1}{c^2}\mathbf{E} \times \mathbf{H}$.[6] However, inside the medium the definition of electromagnetic momentum is more subtle, and different formulations exist.[7] Here we use the Abraham form, and take the momentum density of light inside the medium to be $\mathbf{g} = \frac{1}{c^2}\mathbf{E} \times \mathbf{H}$.

The Lorentz force density can be written

$$\mathbf{f} = (\mathbf{P} \cdot \nabla)\mathbf{E} + \frac{\partial \mathbf{P}}{\partial t} \times \mathbf{B} \tag{2}$$

where $\mathbf{P}$ is the polarisation density*.[8]

Substituting in these expressions for momentum density and Lorentz force density, equation (1) becomes:

$$\frac{1}{c^2}\frac{\partial}{\partial t}\left(\varepsilon_{ijk}E_jH_k\right) + P_j\nabla_jE_i + \varepsilon_{ijk}\dot{P}_jB_k = -\nabla_jT_{ij}, \tag{3}$$

where a dot above a symbol indicates a time derivative. Assuming that the material is nonmagnetic, we have $\mathbf{H} = \frac{1}{\mu_0}\mathbf{B}$, so the left-hand side of equation (3) can be rewritten as:

$$\varepsilon_0\left(\varepsilon_{ijk}\dot{E}_jB_k + \varepsilon_{ijk}E_j\dot{B}_k\right) + P_j\nabla_jE_i + \varepsilon_{ijk}\dot{P}_jB_k. \tag{4}$$

We then use the definition of the displacement field, $\mathbf{D} = \varepsilon_0\mathbf{E} + \mathbf{P}$, to remove the polarisation field from the expression above:

---

* Note that this expression treats the material as a collection of electric dipoles specified by $\boldsymbol{P}$, and the electric part of the Lorentz force is taken as force on the centre of each dipole. An alternative approach would be to consider the Lorentz force on each individual charge, rather than each dipole – the distinction between the two approaches is relevant to the torque calculation of Supplementary section 12.

$$\varepsilon_0\left(\varepsilon_{ijk}\dot{E}_jB_k+\varepsilon_{ijk}E_j\dot{B}_k\right)+P_j\nabla_jE_i+\varepsilon_{ijk}\dot{P}_jB_k$$

$$=\varepsilon_0\left(\varepsilon_{ijk}\dot{E}_jB_k+\varepsilon_{ijk}E_j\dot{B}_k\right)+(D_j-\varepsilon_0E_j)\nabla_jE_i+\varepsilon_{ijk}(\dot{D}_j-\varepsilon_0\dot{E}_j)B_k$$

$$=\varepsilon_0\varepsilon_{ijk}E_j\dot{B}_k+(D_j-\varepsilon_0E_j)\nabla_jE_i+\varepsilon_{ijk}\dot{D}_jB_k. \quad (5)$$

We now use Maxwell's equations $\nabla\times\mathbf{E}=-\dot{\mathbf{B}}$ and $\nabla\times\mathbf{H}=\dot{\mathbf{D}}=\frac{1}{\mu_0}\nabla\times\mathbf{B}$ (where we assume that the medium is non-magnetic):

$$\varepsilon_0\varepsilon_{ijk}E_j\dot{B}_k+(D_j-\varepsilon_0E_j)\nabla_jE_i+\varepsilon_{ijk}\dot{D}_jB_k$$

$$=-\varepsilon_0\varepsilon_{ijk}E_j\varepsilon_{klm}\nabla_lE_m+(D_j-\varepsilon_0E_j)\nabla_jE_i+\frac{1}{\mu_0}\varepsilon_{ijk}B_k\varepsilon_{jlm}\nabla_lB_m. \quad (6)$$

Finally, we use the identity $\varepsilon_{abc}\varepsilon_{ade}=\delta_{bd}\delta_{ce}-\delta_{be}\delta_{cd}$ to write:

$$-\varepsilon_0\varepsilon_{ijk}E_j\varepsilon_{klm}\nabla_lE_m+(D_j-\varepsilon_0E_j)\nabla_jE_i+\frac{1}{\mu_0}\varepsilon_{ijk}B_k\varepsilon_{jlm}\nabla_lB_m$$

$$=-\varepsilon_0(\delta_{il}\delta_{jm}-\delta_{im}\delta_{jl})E_j\nabla_lE_m+(D_j-\varepsilon_0E_j)\nabla_jE_i+\frac{1}{\mu_0}(\delta_{lk}\delta_{im}-\delta_{km}\delta_{il})B_k\nabla_lB_m$$

$$=-\varepsilon_0E_j\nabla_iE_j+D_j\nabla_jE_i+\frac{1}{\mu_0}B_k\nabla_kB_i-\frac{1}{\mu_0}B_k\nabla_iB_k. \quad (7)$$

Our task is now to write the expression (7) as the divergence of a second rank tensor. It is straightforward to show that this is accomplished if we define

$$T_{ij}=-E_iD_j-\frac{1}{\mu_0}B_iB_j+\frac{1}{2}\left(\varepsilon_0E_kE_k+\frac{1}{\mu_0}B_kB_k\right)\delta_{ij}, \quad (8)$$

since $-\nabla_jT_{ij}$ is:

$$-\nabla_jT_{ij}=-\nabla_j\left[-E_iD_j-\frac{1}{\mu_0}B_iB_j+\frac{1}{2}\left(\varepsilon_0E_kE_k+\frac{1}{\mu_0}B_kB_k\right)\delta_{ij}\right]$$

$$=D_j\nabla_jE_i+E_i\nabla_jD_j+\frac{1}{\mu_0}B_i\nabla_jB_j+\frac{1}{\mu_0}B_j\nabla_jB_i-\varepsilon_0E_k\nabla_iE_k-\frac{1}{\mu_0}B_k\nabla_iB_k. \quad (9)$$

Assuming there are no free charges, we can use Maxwell's equations $\nabla \cdot \mathbf{D} = 0$ and $\nabla \cdot \mathbf{B} = 0$, so the equality of equations (7)(7) and (9)(9) immediately follows.

13. Derivation of angular momentum continuity equation inside a medium

Angular momentum $\mathbf{L}$ is the cross product of the position vector $\mathbf{r}$ with the linear momentum $\mathbf{p}$:

$$\mathbf{L} = \mathbf{r} \times \mathbf{p}. \quad (10)$$

Similarly, we can define the angular momentum flux density as the "cross product" of the position vector with the momentum flux tensor.[3] The i-component of the angular momentum flux in the l direction is:

$$M_{li} = \varepsilon_{ijk} r_j T_{kl}. \quad (11)$$

Using the expression derived for the linear momentum flux density in supplementary section 11 (equation (8)), the angular momentum flux density in the medium is given by

$$M_{li} = \varepsilon_{ijk} r_j T_{kl} = \varepsilon_{ijk} r_j \left[\frac{1}{2} \delta_{kl} \left(\varepsilon_0 E_m E_m + \frac{1}{\mu_0} B_m B_m \right) - \varepsilon_0 E_k D_l - \mu_0^{-1} B_k B_l \right]. \quad (12)$$

To obtain a continuity equation for the angular momentum of light, we begin by taking the cross product of the position vector $\mathbf{r}$ and the linear momentum continuity equation (1)(1):

$$\frac{1}{c^2} \varepsilon_{ijk} r_j \frac{\partial}{\partial t} (\mathbf{E} \times \mathbf{H})_k + \varepsilon_{ijk} r_j P_m \nabla_m E_k + \varepsilon_{ijk} r_j \varepsilon_{klm} \dot{P}_l B_m = - \varepsilon_{ijk} r_j \nabla_l T_{kl}. \quad (13)$$

The first term in equation (13) represents the time derivative of the angular momentum density $\frac{1}{c^2}\mathbf{r} \times (\mathbf{E} \times \mathbf{H})$. The second two terms are $\mathbf{r} \times \mathbf{f}$, where $\mathbf{f}$ is the Lorentz force density given in equation (2), and so these correspond to a torque density. However, this is not the total torque density. The force density was defined as the force acting on the centre of each electric dipole, and therefore the torque density derived from this does not include the torque on each individual dipole about its own centre. We might expect an extra torque, with a form like $\boldsymbol{\tau}_{orienting} = \mathbf{P} \times \mathbf{E}$, which would act to orient each dipole to align with the electric field.

Finally, we note that the term on the right-hand side is not quite equal to the divergence of the angular momentum flux density. The divergence of M is equal to the divergence of the cross product of r and T, but so far the right-hand side of equation (13) is the cross product of r with the divergence of T. We can see that the divergence of M will have an extra contribution, due to the product rule when the divergence is taken:

$$\nabla_l M_{li} = \nabla_l \left(\varepsilon_{ijk} r_j T_{kl} \right) = \varepsilon_{ijk} T_{kl} \nabla_l (r_j) + \varepsilon_{ijk} r_j \nabla_l (T_{kl}). \quad (14)$$

The expression $\varepsilon_{ijk} T_{kl} \nabla_l (r_j)$ becomes

$$\varepsilon_{ijk} T_{kl} \nabla_l (r_j) = \varepsilon_{ijk} T_{kl} \delta_{lj}$$

$$= \varepsilon_{ijk} \delta_{lj} \Big(-E_k D_l - \frac{1}{\mu_0} B_k B_l + \frac{1}{2} \Big(\varepsilon_0 E_m E_m + \frac{1}{\mu_0} B_m B_m \Big) \delta_{kl} \Big)$$

$$= \varepsilon_{ijk} \Big(-E_k D_j - \frac{1}{\mu_0} B_k B_j + \frac{1}{2} \Big(\varepsilon_0 E_m E_m + \frac{1}{\mu_0} B_m B_m \Big) \delta_{kj} \Big). \qquad (15)$$

Because $\varepsilon_{ijk} \delta_{kj} = 0$, the part involving E^2 and B^2 vanishes, and because ε_{ijk} is antisymmetric, the contraction with the symmetric tensor $B_k B_j$ also vanishes. We can then use $\mathbf{D} = \varepsilon_0 \mathbf{E} + \mathbf{P}$ to write:

$$\varepsilon_{ijk} T_{kl} \nabla_l (r_j) = -\varepsilon_{ijk} E_k \big(\varepsilon_0 E_j + P_j \big) = -\varepsilon_{ijk} E_k P_j, \qquad (16)$$

where we have again used the antisymmetry of ε_{ijk} to write $\varepsilon_{ijk} E_k E_j = 0$. Equation (14) therefore becomes:

$$\nabla_l M_{li} = \varepsilon_{ijk} r_j \nabla_l T_{kl} - (\mathbf{P} \times \mathbf{E})_i. \qquad (17)$$

We can therefore see that if we add $(\mathbf{P} \times \mathbf{E})_i$ to both sides of equation (13), we obtain the continuity equation:

$$\frac{1}{c^2} \varepsilon_{ijk} r_j \frac{\partial}{\partial t} (\mathbf{E} \times \mathbf{H})_k + \varepsilon_{ijk} r_j P_m \nabla_m E_k + \varepsilon_{ijk} r_j \varepsilon_{klm} \dot{P}_l B_m + \varepsilon_{ijk} P_j E_k = -\nabla_l M_{li}. \qquad (18)$$

Equation (18) is the continuity equation for angular momentum inside the medium: the divergence of the angular momentum flux density on the right-hand side is equated to the local rate of change of angular momentum on the left. This rate of change is given by the time derivative of the optical angular momentum density (both the torque due to the Lorentz force density $\mathbf{r} \times (\mathbf{P} \cdot \nabla)\mathbf{E} + \frac{\partial \mathbf{P}}{\partial t} \times \mathbf{B}$, and also the torque on each dipole about its own centre, $\mathbf{P} \times \mathbf{E}$).

14. Calculation of net forces and torques imparted on a material

Recall that the continuity equation for linear momentum of light is given by (Supplementary Section 11):

$$\frac{1}{c^2}\frac{\partial}{\partial t}\left(\varepsilon_{ijk}E_jH_k\right) + P_j\nabla_jE_i + \varepsilon_{ijk}\dot{P}_jB_k = -\nabla_jT_{ij}, \qquad (19)$$

where T_{ij} is the momentum flux density given in equation (8). If an experiment is performed with a continuous source of monochromatic light, then it is possible to time-average the continuity equation over a cycle. Under this averaging, the term corresponding to the time derivative of the Poynting vector vanishes, and we are therefore able to equate the divergence of the momentum flux density with the force on the dielectric,

$$P_j\nabla_jE_i + \varepsilon_{ijk}\dot{P}_jB_k = -\nabla_jT_{ij}. \qquad (20)$$

This can be rewritten with the divergence theorem to find the force on a volume in terms of the integral of the momentum flux through the surface enclosing the volume,

$$\iiint force_i\, dV = -\oint T_{ij}n_j dS\,, \qquad (21)$$

where n_j is a unit vector in the direction of the surface element dS. Therefore, the force imparted by the beam at an interface can be calculated by considering the surface integral of T_{ij} over a surface enclosing the interface; this could, for example, be the difference in momentum fluxes through a plane just before the interface and one just after.

Similarly, a net torque can be obtained from the surface integral of the angular momentum flux density in the same way as a force can be obtained from the linear momentum. If a beam is propagating in the z direction normal to an interface, then the torque about this propagation direction will be given by the difference in the fluxes of the z component of angular momentum through two planes on either side of the interface. The relevant component of the angular momentum flux density tensor, to be evaluated on either side of the interface, is:

$$M_{zz} = \varepsilon_{zjk}r_jT_{kz} = \varepsilon_{zjk}r_j\left[-E_kD_z - \frac{1}{\mu_0}B_kB_z + \frac{1}{2}\left(\varepsilon_0E_mE_m + \frac{1}{\mu_0}B_mB_m\right)\delta_{kz}\right]$$

$$= y(E_xD_z + \mu_0^{-1}B_xB_z) - x\left(E_yD_z + \mu_0^{-1}B_yB_z\right) \qquad (22)$$

where we have made use of the fact that $\varepsilon_{azz} = 0$. This quantity is in essence the cross product of position $\mathbf{r}$ and the momentum flux density.

We now compute the cycle-average of equation (22) for monochromatic fields. We begin by expressing the real electric and magnetic field components E_i and B_j in terms of complex fields $\mathcal{E}_i$ and $\mathcal{B}_j$:

$$E_i = Re\{\mathcal{E}_i\} = Re\{\tilde{E}_i \exp(-i\omega t)\} \tag{23}$$

$$B_j = Re\{\mathcal{B}_j\} = Re\{\tilde{B}_j \exp(-i\omega t)\}, \tag{24}$$

with complex amplitudes $\tilde{E}_i$ and $\tilde{B}_j$. Maxwell's equations in the medium in the absence of sources are:

$$\nabla \cdot \mathbf{B} = 0. \tag{25}$$

$$\nabla \cdot \mathbf{D} = 0. \tag{26}$$

$$\nabla \times \mathbf{E} + \frac{\partial \mathbf{B}}{\partial t} = 0. \tag{27}$$

$$\nabla \times \mathbf{H} - \frac{\partial \mathbf{D}}{\partial t} = 0. \tag{28}$$

Note $\mathbf{D} = \varepsilon \mathbf{E}$ and $\mathbf{B} = \mu \mathbf{H}$.

In terms of the complex fields, Maxwell's equations become

$$\nabla \cdot \boldsymbol{\mathcal{B}} = 0, \tag{29}$$

$$\nabla \cdot \boldsymbol{\mathcal{D}} = 0, \tag{30}$$

$$\nabla \times \boldsymbol{\mathcal{E}} - i\omega \boldsymbol{\mathcal{B}} = 0, \tag{31}$$

$$\nabla \times \boldsymbol{\mathcal{H}} + i\omega \boldsymbol{\mathcal{D}} = 0. \tag{32}$$

From the last two equations we obtain:

$$\tilde{B}_j = \frac{1}{i\omega} \varepsilon_{jkl} \frac{\partial}{\partial r_k} \tilde{E}_l, \tag{33}$$

$$\tilde{D}_i = -\frac{1}{i\omega} \varepsilon_{ikl} \frac{\partial}{\partial r_k} \tilde{H}_l. \tag{34}$$

The cycle-averaged angular momentum flux density is given by:

$$\overline{M}_{zz} = \frac{1}{2} Re\{y(\tilde{E}_x\tilde{D}_z^* + \mu_0^{-1}\tilde{B}_x\tilde{B}_z^*) - x(\tilde{E}_y\tilde{D}_z^* + \mu_0^{-1}\tilde{B}_y\tilde{B}_z^*)\}$$

$$= \frac{1}{2} Re\{(y\tilde{E}_x - x\tilde{E}_y)\tilde{D}_z^* + \mu_0^{-1}(y\tilde{B}_x - x\tilde{B}_y)\tilde{B}_z^*\}$$

$$= \frac{1}{2} Re\left\{(y\tilde{E}_x - x\tilde{E}_y)\frac{1}{i\omega}\left(\frac{\partial}{\partial x}\tilde{H}_y^* - \frac{\partial}{\partial y}\tilde{H}_x^*\right) + \mu_0^{-1}(y\tilde{B}_x - x\tilde{B}_y)\frac{1}{i\omega}\left(\frac{\partial}{\partial y}\tilde{E}_x^* - \frac{\partial}{\partial x}\tilde{E}_y^*\right)\right\}$$

$$= \frac{1}{2\omega} Re\left\{-i\left[(y\tilde{E}_x - x\tilde{E}_y)\left(\frac{\partial}{\partial x}\tilde{H}_y^* - \frac{\partial}{\partial y}\tilde{H}_x^*\right) + \mu_0^{-1}(y\tilde{B}_x - x\tilde{B}_y)\left(\frac{\partial}{\partial y}\tilde{E}_x^* - \frac{\partial}{\partial x}\tilde{E}_y^*\right)\right]\right\} \quad (35)$$

The angular momentum (AM) flux is then obtained from the surface integral of the angular momentum flux density $\overline{M}_{zz}$:

$$\begin{aligned}(AM\,flux)_{zz} = \frac{1}{2\omega} Re\Big\{-i\iint \Big[(y\tilde{E}_x - x\tilde{E}_y)\left(\frac{\partial}{\partial x}\tilde{H}_y^* - \frac{\partial}{\partial y}\tilde{H}_x^*\right) \\ + \mu_0^{-1}(y\tilde{B}_x - x\tilde{B}_y)\left(\frac{\partial}{\partial y}\tilde{E}_x^* - \frac{\partial}{\partial x}\tilde{E}_y^*\right)\Big]dxdy\Big\}\end{aligned} \quad (36)$$

For a linearly-polarised b eam (*i.e*. no spin angular momentum), the AM flux equals to the orbital angular momentum (OAM) flux.

15. Supplementary numerical simulations of angular momentum flux in WS_2

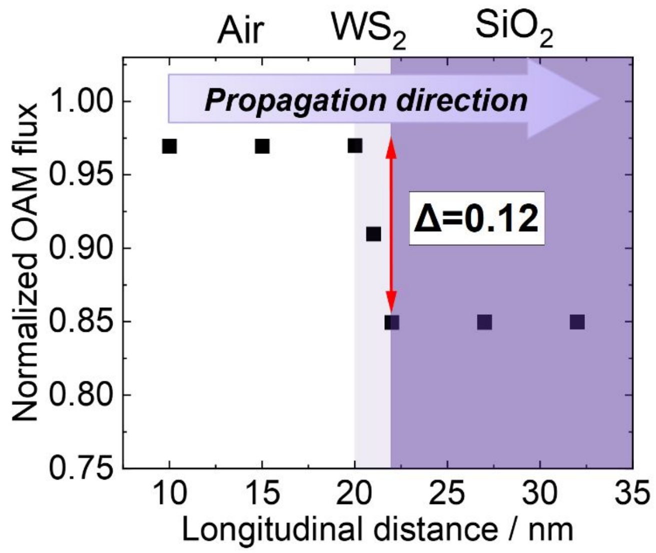

Figure S20. **Simulated orbital angular momentum flux as an LG beam ($l = 4$) propagates through air, WS_2 and SiO_2, normalised by the orbital angular momentum flux of the beam in air.**

References

1. Padgett, M. J.; Allen, L., The Poynting Vector in Laguerre-Gaussian Laser Modes. *Optics Communications* **1995,** *121* (1-3), 36-40.
2. Padgett, M. J.; Miatto, F. M.; Lavery, M. P. J.; Zeilinger, A.; Boyd, R. W., Divergence of an orbital-angular-momentum-carrying beam upon propagation. *New Journal of Physics* **2015,** *17* (2), 023011.
3. Barnett, S. M., Optical angular-momentum flux. *Optics B: Quantum and Semiclassical Optics* **2002,** *4* (2), S7.
4. McCreary, K. M.; Hanbicki, A. T.; Singh, S.; Kawakami, R. K.; Jernigan, G. G.; Ishigami, M.; Ng, A.; Brintlinger, T. H.; Stroud, R. M.; Jonker, B. T., The Effect of Preparation Conditions on Raman and Photoluminescence of Monolayer WS2. *Sci Rep* **2016,** *6*, 35154.
5. Wei, K.; Liu, Y.; Yang, H.; Cheng, X. A.; Jiang, T., Large range modification of exciton species in monolayer WS2. *Appl Optics* **2016,** *55* (23), 6251-6255.
6. Jackson, J. D., *Classical electrodynamics*. Third edition. New York : Wiley, [1999] ©1999: 1999.
7. Barnett, S. M.; Loudon, R., The enigma of optical momentum in a medium. *Philosophical Transactions of the Royal Society A: Mathematical, Physical and Engineering Sciences* **2010,** *368* (1914), 927-939.
8. Barnett, S. M.; Loudon, R., On the electromagnetic force on a dielectric medium. *Journal of Physics B: Atomic, Molecular and Optical Physics* **2006,** *39* (15), S671.
